# Antibacterial Activity of Fluorobenzoylthiosemicarbazides and Their Cyclic Analogues with 1,2,4-Triazole Scaffold

**DOI:** 10.3390/molecules26010170

**Published:** 2020-12-31

**Authors:** Urszula Kosikowska, Monika Wujec, Nazar Trotsko, Wojciech Płonka, Piotr Paneth, Agata Paneth

**Affiliations:** 1 Department of Pharmaceutical Microbiology, Faculty of Pharmacy, Medical University of Lublin, Chodźki 1, 20-093 Lublin, Poland; urszula.kosikowska@umlub.pl; 2 Department of Organic Chemistry, Faculty of Pharmacy, Medical University of Lublin, Chodźki 4a, 20-093 Lublin, Poland; monika.wujec@umlub.pl (M.W.); nazar.trotsko@umlub.pl (N.T.); 3 FQS-Fujitsu Poland, Parkowa 11, 33-332 Kraków, Poland; w.plonka@fqs.pl; 4 Institute of Applied Radiation Chemistry, Faculty of Chemistry, Lodz University of Technology, Żeromskiego 116, 90-924 Lodz, Poland; piotr.paneth@p.lodz.pl

**Keywords:** thiosemicarbazides, 1,2,4-triazoles, antibacterial activity, *S. aureus* clinical isolates, SAR/QSAR analysis

## Abstract

The development of drug-resistant bacteria is currently one of the major challenges in medicine. Therefore, the discovery of novel lead structures for the design of antibacterial drugs is urgently needed. In this structure–activity relationship study, a library of *ortho-*, *meta-*, and *para*-fluorobenzoylthiosemicarbazides, and their cyclic analogues with 1,2,4-triazole scaffold, was created and tested for antibacterial activity against Gram-positive bacteria strains. While all tested 1,2,4-triazoles were devoid of potent activity, the antibacterial response of the thiosemicarbazides was highly dependent on substitution pattern at the N4 aryl position. The optimum activity for these compounds was found for trifluoromethyl derivatives such as **15a**, **15b**, and **16b**, which were active against both the reference strains panel, and pathogenic methicillin-sensitive and methicillin-resistant *Staphylococcus aureus* clinical isolates at minimal inhibitory concentrations (MICs) ranging from 7.82 to 31.25 μg/mL. Based on the binding affinities obtained from docking, the conclusion can be reached that fluorobenzoylthiosemicarbazides can be considered as potential allosteric d-alanyl-d-alanine ligase inhibitors.

## 1. Introduction

A huge number of drugs and clinical candidates for development contain halogen atoms. For a long time, predominantly only the steric and hydrophobic factors of halogens were considered when trying to exploit their role in ligand recognition and target–ligand binding complex stabilization. Today, the ability of halogens to form favorable intermolecular interactions, such as halogen bonds, hydrogen bonds, and multipolar interactions that significantly contribute to the stability of target–ligand complexes has been well recognized, and widely utilized in the rational design of new therapeutics against clinically significant targets [1,2,3,4]. According to an analysis by Hernandes and co-workers [5], the majority of halogenated drugs are fluorine drugs (57%), followed by chlorine ones (38%), while those with bromine are rare (4%), and only a few iodinated drugs are known (1%) due to their relatively instability, caused by the high polarizability of the C–I bond.

Regarding the fluorinated drugs and drug candidates, fluorine substitutions have been extensively investigated in drug research and there are some important reasons that justify their predominance. For instance, several studies have documented that selective introduction of a trifluoromethyl group or even a single fluorine atom into a in a key position of a therapeutic molecule significantly enhances its pharmacokinetic and physicochemical properties, such as improved metabolic stability and membrane permeability [6]. The increased binding affinity of fluorine-containing drug candidates to molecular targets has also been proved in several cases [7,8,9,10,11,12,13,14,15,16,17,18,19]. Currently, several fluorinated pharmaceuticals are widely used in clinical practice, and nearly one-third of the 100 top-selling drugs are organofluorine compounds. The most common among these are fluoroquinolone antibiotics, antimalarials, antidepressants, antivirals, anesthetics, anticancers, cholesterol-lowering drugs, anti-inflammatory drugs, and asthma drugs, and several reviews on potential therapeutic applications of fluorinated molecules have been published in recent years [4,20,21,22]. For these reasons, we explored the antibacterial potential within the structure–activity relationship of a library of 1-fluorobenzoyl-4-aryl(alkyl)thiosemicarbazides. Previously, we had searched for the N1 substituents which would improve the antibacterial potency of thiosemicarbazide-based compounds and had found that the fluorobenzoyl group might constitute a particularly useful series [23]. Other research groups also confirmed the thiosemicarbazide structure as a novel scaffold for potential antibacterial agents [24,25,26,27,28,29,30,31,32,33], although a systematic survey of the effects of the N1 and N4 terminal substituents on antibacterial potency has not been presented thus far. Thus, in an attempt to more closely define the factors controlling the potency of these compounds, we prepared three series, with *ortho-*, *meta-*, and *para*-fluorobenzoyl groups, and determined their antibacterial activity in vitro with the structure–activity relationship. In carrying out this study of the structure–activity relationship, our strategy was four-stage. First, we sought to investigate the effect of the aromatic fluorine substitution (*ortho*, *meta*, and *para*) and the N4 alkyl and aryl groups. Second, we sought to extend the antibacterial assay on methicillin-sensitive and methicillin-resistant *Staphylococcus aureus* clinical isolates. Third, we set out to alter the linear structure of the thiosemicarbazide by the synthesis of cyclic analogues with a 1,2,4-triazole scaffold, and to compare these with the corresponding thiosemicarbazide counterparts. Finally, our results of docking our analogues to the allosteric site of d-alanyl-d-alanine ligase concurred with the pioneering reports made by Ameryckx et al. [24,25] on the molecular basis of the antibacterial activity of benzoylthiosemicarbazides.

## 2. Results and Discussion

### 2.1. Synthetic Protocols

**Scheme 1:**
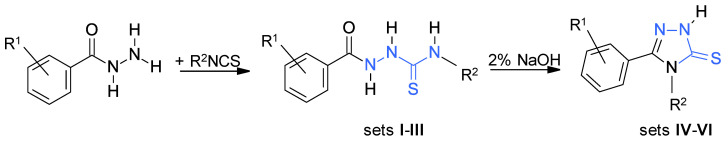
Synthetic route for 1-fluorobenzoyl-4-aryl(alkyl)thiosemicarbazides (sets **I**–**III**) and 1,2,4-triazole-3-thiones (sets **IV**–**VI**). **R^1^** = *o*-F (set **I**-**a,** set **IV**-**at**), *m*-F (set **II**-**b**, **set V**-**bt**), *p*-F (set **III**-**c**, set **VI**-**ct**). **R^2^** = Pr (**1**), Bu (**2**), Ph (**3**), 1-naph (**4**), *m*-tol (**5**), *p*-tol (**6**), *m*-F (**7**), *p*-F (**8**), *m*-Cl (**9**), *p*-Cl (**10**), *m*-Br (**11**), *p*-Br (**12**), *m*-I (**13**), *p*-I (**14**), *m*-CF_3_ (**15**), *p*-CF_3_ (**16**).

The synthesis of *ortho*-, *meta*-, and *para*-fluorobenzoylthiosemicarbazides (sets **I**–**III**) and their cyclic analogues with a 1,2,4-triazole-5-thione scaffold (sets **IV**–**VI**) was performed according to a well-established synthetic route, described elsewhere [23,34] (Scheme 1). The reaction of the corresponding fluorobenzoylhydrazide with related alkyl/aryl isothiocyanate in boiling ethanol gave 1-fluorobenzoyl-4-aryl/(alkyl)thiosemicarbazides (sets **I**–**III**) in 40–90% yields. Resulting compounds were cyclized in boiling aqueous sodium hydroxide to give corresponding 1,2,4-triazole-3-thiones (sets **IV**–**VI**), which were obtained in 48–90% yields.

### 2.2. Antibacterial Screening 

The thiosemicarbazides (sets **I**–**III**) were evaluated for their antibacterial activity against a panel of the reference Gram-positive bacteria using the standard two-fold serial microdilution assay described by the Clinical and Laboratory Standards Institute [35]. The results for the minimum inhibitory concentrations (MICs) are reported in Table 1. Commercially available antibiotic cefuroxime was used as the reference control.

**Figure 1:**
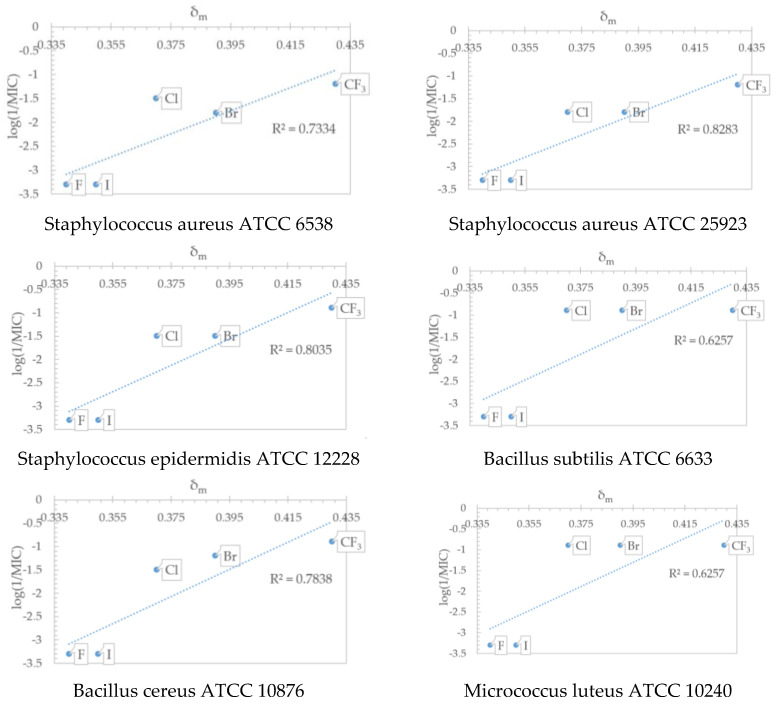
Plot of values of log(1/MIC) for bacterial strains determined experimentally for the compounds of set **I** with *meta* electron-withdrawing substitution (**7a**, **9a**, **11a**, **13a**, **15a**), versus the appropriate values of the σ_m_ Hammett electronic substituent constant.

A few general conclusions arose from the antibacterial assay, which can be summarized as follows. The most important is that within set **I** of *ortho*-fluorobenzoylthiosemicarbazides **1a**–**16a**, the compounds that bear substituents at the N4 aryl position that are electron acceptors were more active than those that are either unsubstituted or bear electron donors. Electron-withdrawing substituents in the *meta* position (**7a**, **9a**, **11a**, **13a**, **15a**) generally increased the antibacterial potency, and within *meta* electron-withdrawing compounds the activity follows the trend: *meta*-CF_3_ **15a** (MIC 7.82–15.63 µg/mL; 0.02–0.04 mM) > *meta*-Cl **9a** (MIC 7.82–62.5 µg/mL; 0.02–0.19 mM) ~ *meta*-Br **11a** (MIC 7.82–62.5 µg/mL; 0.02–0.17 mM) > *meta*-F **7a** = *meta*-I **13a** (n.a.; MICs at 2000 µg/mL; 6.51 and 4.82 mM, respectively). The assessment of the results of the *meta* electron-withdrawing compounds was made in three terms: (i) an electronic one, expressed by the Hammett substituent constant σ_m_ [37,38,39] and inductive substituent constant σ_I_; (ii) a steric, expressed by the molar refractivity *MR,* the Taft’s constant *Es* [39,40,41,42], and the Charton steric parameter *σ_ν_* [39,43,44,45,46]; and (iii) a hydrophobic, expressed by cLog*P* and π, the Hansch substituent constant [39,47], and led to the conclusion that the single most important physicochemical property that could explain the variance in the MICs of these compounds is the Hammett substituent constant σ_m_. Indeed, as presented in Figure 1, a good correlation exists between the MICs of the *meta*-substituted compounds **7a**, **9a**, **11a**, **13a**, **15a,** and the σ_m_ constants, whereas no correlation could be found between their MICs and steric or hydrophobic parameters. Comparative σ_m_, σ_I_, σ_ν_, *MR*, *Es*, π, and cLog*P* values are listed in Table S1. The fact that the improvement in activity of these compounds is generally correlated with the Hammett substituent constant σ_m_ (F < I < Cl < Br < CF_3_) suggests that the electronic properties of the *meta* electron withdrawing substituents control the antibacterial activity. While it remains unclear what role, if any, the electronegativity of the *meta* substituents plays in antibacterial activity, possible interpretations of the correlation between the antibacterial activity of **7a**, **9a**, **11a**, **13a**, **15a,** and σ_m_ are therefore that: (i) antibacterial activity increases with decreasing electron density on the thiosemicarbazide scaffold because enhancement in binding of the thiosemicarbazide to the molecular target is created through the increased positive charge character of the N4 aryl ring or whole thiosemicarbazide scaffold; or (ii) antibacterial activity increases with decreasing pKa of the thiosemicarbazide core (NH-NH-C(=S)-NH), because the compound must be deprotonated to be active (deprotonation hypothesis) [48]. A combination of both effects is possible. A second important observation is that although the steric substituent constants, such as the Taft *Es* constant or Charton *σ_ν_* parameter, do not directly explain the trend in antibacterial activity of *meta* compounds **7a**, **9a**, **11a**, **13a**, and **15a**, the most potent antibacterial *meta*-CF_3_ **15a** proved to possess the lowest Taft *Es* constant and the highest Charton *σ_ν_* parameter, thus suggesting that the bulkiness effect of the CF_3_ substituent induced an improvement in antibacterial activity. The last but not least observation that arose from the antibacterial assay of the thiosemicarbazides of **set I,** is that substitutions in the *para* position of the N4 phenyl ring abolished activity (Table 1, compounds **6a**, **8a**, **10a**, **12a**, and **14a**). The only exceptions were *para*-CF_3_ **16a** and *para*-Cl **10a,** which inhibited the growth of *Bacillus cereus* at a concentration similar to cefuroxime, and *Micrococcus luteus* at the MIC of 7.82 µg/mL (0.02 mM). The fact that **10a** and **16a** still possess antibacterial activity suggest that although the protein binding pocket provides sufficient space for additional groups at the *para* position, the combination of both steric and specific, i.e., hydrophobic and electrostatic or intermolecular interactions might be the most prominent factor regulating the activity of the *para* substituted thiosemicarbazides of set **I**.

For the *meta*-fluorobenzoylthiosemicarbazides of set II (**1b**–**16b**), a reverse trend of antibacterial activity was observed compared to *ortho* set I. Clearly, within set II, *para*-Cl **10b** with MICs in the range of 7.82–15.63 µg/mL (0.02–0.19 mM) against *Staphylococcus spp*., *B. subtilis*, and *M. luteus* showed a clear preference for the potency whereas the antibacterial activity of *para*-CF_3_ **16b** was comparable to that of *meta*-CF_3_ **15b**. Furthermore, *meta*-I **13b**, in contrast to inactive *meta*-I **13a**, was able to inhibit the growth of *S. aureus*, *B. subtilis*, *B. cereus*, and *M. luteus* at the concentration of 15.63 µg/mL (0.04 mM), whereas the antibacterial activity of *meta*-Cl **9b** and *meta*-Br **11b** against *S. aureus* strains was two to four times higher compared to their isomers, ***9a*** and ***11a**,* respectively. Clearly, within set II, *meta*-Cl and *para*-Cl compounds (**9b**, **10b**), and *meta*-CF_3_ and *para*-CF_3_ compounds (**15b**, **16b**), proved to be the most effective. No important contribution(s), however, of electronic, steric, and/or hydrophobic factors on antibacterial trend within set **II** was found.

Finally, the antibacterial activity of the *para*-fluorobenzoylthiosemicarbazides of set **III** (**1c**–**16c**) was tested. Although these compounds were generally less effective than their *ortho* (set **I**) and *meta* (set **II**) isomers, the efficiency of the compounds with chloro (**9c**, **10c**), bromo (**11c**, **12c**), and iodo (**13c**, **14c**) substitutions against *B. cereus* was still close to cefuroxime. In addition, these compounds with MICs at 15.63 µg/mL (0.05 mM for **9c**, **10c,** and 0.04 mM for **11c**, **12c**, **13c**, **14c**) showed good inhibitory action against *M. luteus*. The presence of other substituents, both an electron withdrawing (CF_3_, F) and an electron donating (CH_3_), reduced potency, whereas the presence of a propyl, butyl, phenyl, or naphthyl group abolished activity; similarly to the antibacterial trends observed for sets **I** and **II**.

To confirm antibacterial potential of the most potent thiosemicarbazides, **15a**, **16a**, **9b**, **10b**, **11b**, **15b**, **16b**, these compounds were subsequently assayed against a panel of pathogenic methicillin-sensitive and methicillin-resistant *Staphylococcus aureus* clinical isolates. As indicated from the data collected in Table 2, the best antibacterial response, with MICs in the range of 3.91–15.63 µg/mL, was noted for trifluoromethyl derivatives; *meta* **15a** (0.02–0.04 mM) and *para* **16b** (0.04–0.09 mM). *Meta* trifluoromethyl derivative **15b** showed an inhibitory action at a slightly higher concentration (MICs 7.82–31.25 µg/mL; 0.04–0.08 mM), while with the remaining compounds activity was lost.

Since SAR analysis of the fluorobenzoylthiosemicarbazides (sets **I**–**III**) seemed to be more complicated than we expected, as mentioned above, we also prepared their cyclic analogues with a 1,2,4-triazole-3-thione scaffold (sets **IV**–**VI**), and submitted them to antimicrobial assay to determine the role, if any, of the thiosemicarbazide core NH-NH-C(=S)-NH on antibacterial activity. As shown in Table 3, all tested triazoles showed much lower antibacterial potency compared to their acyclic precursors (e.g., **15a** vs. **15at**) or were even inactive (e.g., **13b** vs. **13bt**). It is important to note, that none of them showed potent antibacterial activity. Thus, these results confirmed that, although the linear NH-NH-C(=S)-NH core is a key structural element for antibacterial activity, the electronic effect of the N1 and N4 substituents on the whole thiosemicarbazide skeleton is equally important. It seems equally possible, however, that the N1 and N4 substituents could lead to structural benefits, either by providing favorable conformation of the thiosemicarbazide in the molecular target binding site, or by providing favorable intermolecular contacts. Of course, a combination of both electronic and steric effects is also possible. Another important observation is that the hydrophobic factor cLog*P,* alone, had a negligible influence on the antibacterial response of the tested thiosemicarbazides and *s*-triazoles.

### 2.3. Assessing Bactericidal/Bacteriostatic Characteristics 

**Figure 2:**
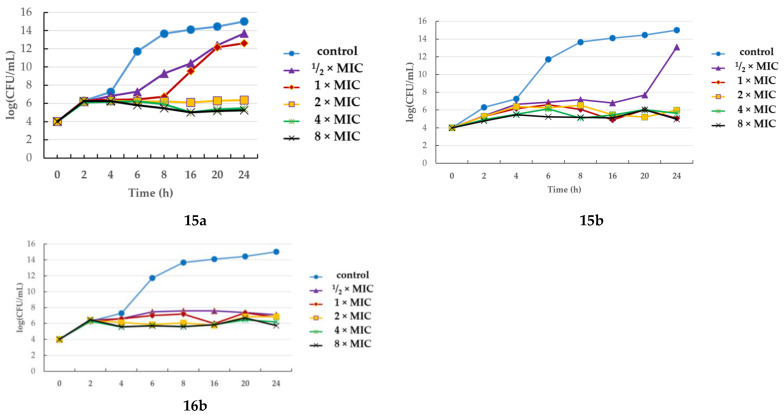
Time-kill kinetics of **15a**, **15b**, and **16b** against *S. aureus* ATCC 25923 over a period of 24 h. Control—*S. aureus* ATCC 25923 growth without tested compounds.

Although the molecular mechanism of the antibacterial activity of thiosemicarbazides is still unknown, their inhibitory action towards bacterial topoisomerases has been proposed as one possible explanation [49,50,51]. Experiments aimed at identifying possible additional targets of these compounds are currently ongoing, but so far the only conclusive results have been yielded by Ameryckx et al., for *ortho*-hydroxybenzoylthiosemicarbazides [24,25]. According to these results, d-alanyl-d-alanine ligase (Ddl) might be considered as a potential bacterial target for the thiosemicarbazides, with the N1 *ortho*-hydroxybenzoyl substitution and subsequent time kill assay for representative analogue, with 2,3-dichlorophenyl group at the N4 position (**PR**), proving that it acts as a bactericidal agent. Generally, when considering the molecular mechanism by which antibacterial agents control microorganisms, the mode of their action can also be classified according to whether they lead to the death of the microbe (bactericidal action), or inhibit its growth (bacteriostatic action). Thus, to distinguish whether our thiosemicarbazides were bactericidal or bacteriostatic in nature, we next submitted them to a minimal bactericidal concentration (MBC) assay. Upon analysis, all compounds were found to be bacteriostatic (MBC/MIC >4) toward all bacteria strains tested (data not shown). Subsequent results from the concentration dependent time-kill assay, for the most potent antibacterial agents **15a**, **15b**, **16b** against *S. aureus* ATCC 25923 over a period of 24 h, confirmed this observation (Figure 2). A concentration-dependent and time-dependent bacteriostatic activity was found for all the compounds tested, even at a high concentration of 4 × MIC or 8 × MIC. Bactericidal activity, defined as a reduction of at least 99.9% of the total count of CFU/mL in the original inoculum within a period of 24 h, was not observed.

As a general rule, antibacterial agents that disrupt the cell wall or cell membrane, or interfere with essential enzymes are often bactericidal, whereas those that inhibit protein synthesis, e.g., bacterial topoisomerases, or interfere with metabolic processes tend to be bacteriostatic [52,53,54]. Taking this fact into account, the results of the MBC assay are not surprising. In fact, in our previous studies [23], on the thiosemicarbazides **9a** and **10a,** we showed that their inhibitory action against *S. aureus* DNA gyrase was very weak; compound **10a** was able to inhibit ~50% of *S. aureus* DNA gyrase activity at as high a concentration as 100 µg/mL, whereas the inhibitory activity of **9a** at this concentration was even lower (~33%). Collectively, the results of the MBC assay further confirm the assumption that, for our bacteriostatic thiosemicarbazides of sets **I**–**III,** bacterial topoisomerases are not primary factors contributing to their antibacterial activity.

### 2.4. Thiol−ThioneTautomerismintheThiosemicarbazides

In an attempt to extract a general background of the relationship between the antibacterial activity and physicochemical properties of the tested thiosemicarbazides and *s*-triazoles, we also performed a QSAR analysis. Before experiments, however, the relative stabilities of the thiosemicarbazide tautomeric forms were studied, based on computational analysis.

**Figure 3:**
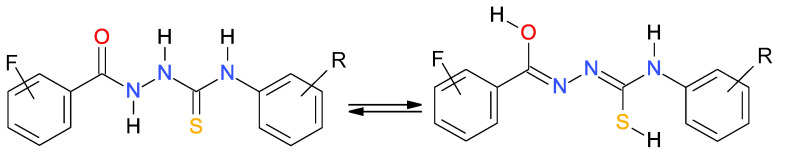
Thione-thiole tautomeric forms of the thiosemicarbazides.

As is known, the proton transfers influence conformational and electronical changes in a molecule, and thus induce changes in its flexibility, polarity, lipophilicity, and acceptor–donor capacity. All these factors have a significant impact on biological activity, including cell membrane permeability, metal complexation, and inter- and intra-molecular interactions. Since the thiosemicarbazides studied here formally can exist in tautomeric thione (C=S) and thiole (C–S) forms [24,25,55,56] (Figure 3), we evaluated theoretically their relative Gibbs free energies. For this purpose, geometries of the thione and thiole forms of the thiosemicarbazides were optimized at the DFT level of theory, using the ωB97X-D functional [57] expressed in the def2-TZVP basis set [58], as implemented in the Gaussian16 program [59]. Vibrational analysis was used to ensure that the optimized geometry corresponded to a stationary point representing a minimum on the potential energy surface (3n − 6 real vibrations). A SMD continuum solvent model [60] was used to model the aqueous solution. Our results indicated that the thiosemicarbazides in the thiole tautomeric form were less stable by about 21.8 kcal/mol, and thus their presence in the aqueous solution can be disregarded. The relative stability in the tautomers of *s*-triazole was studied previously, and it was established that thione tautomer is also the energetically most stable form [61].

### 2.5. QSAR Analysis

**Figure 4:**
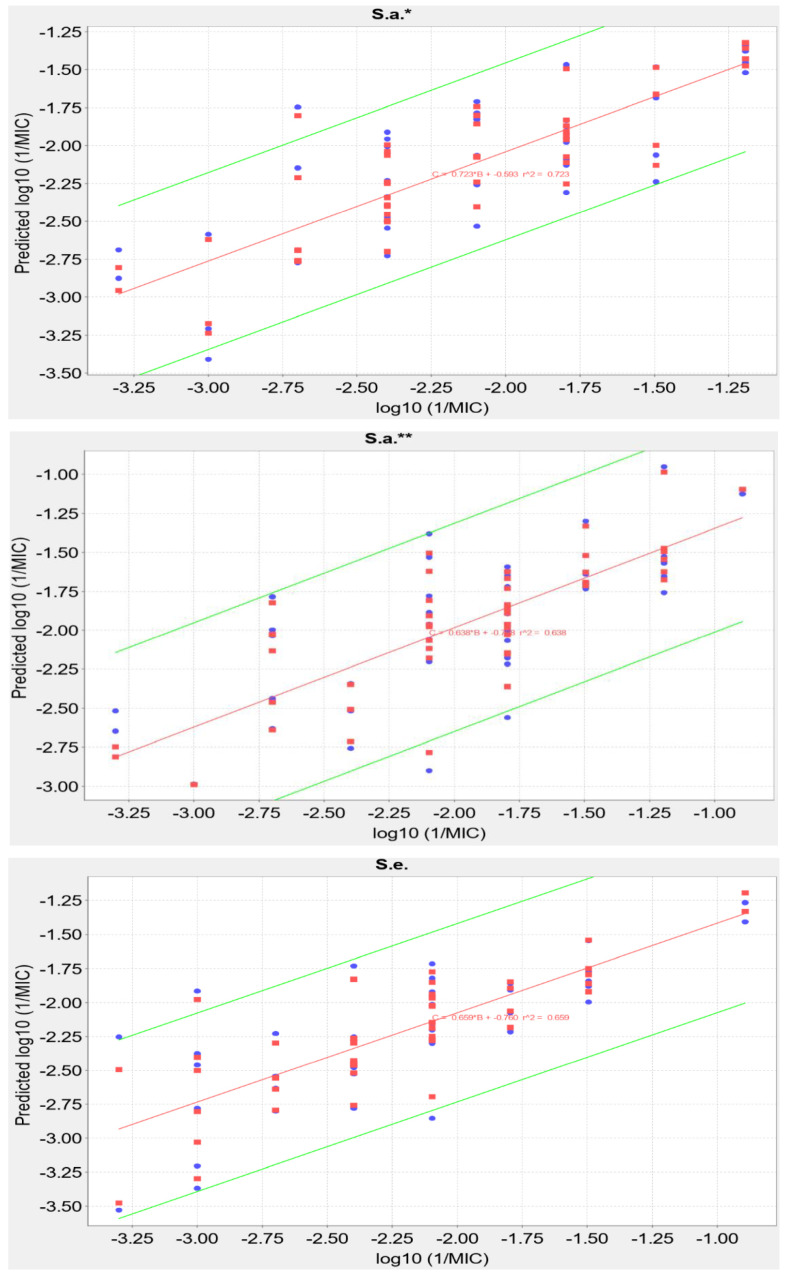

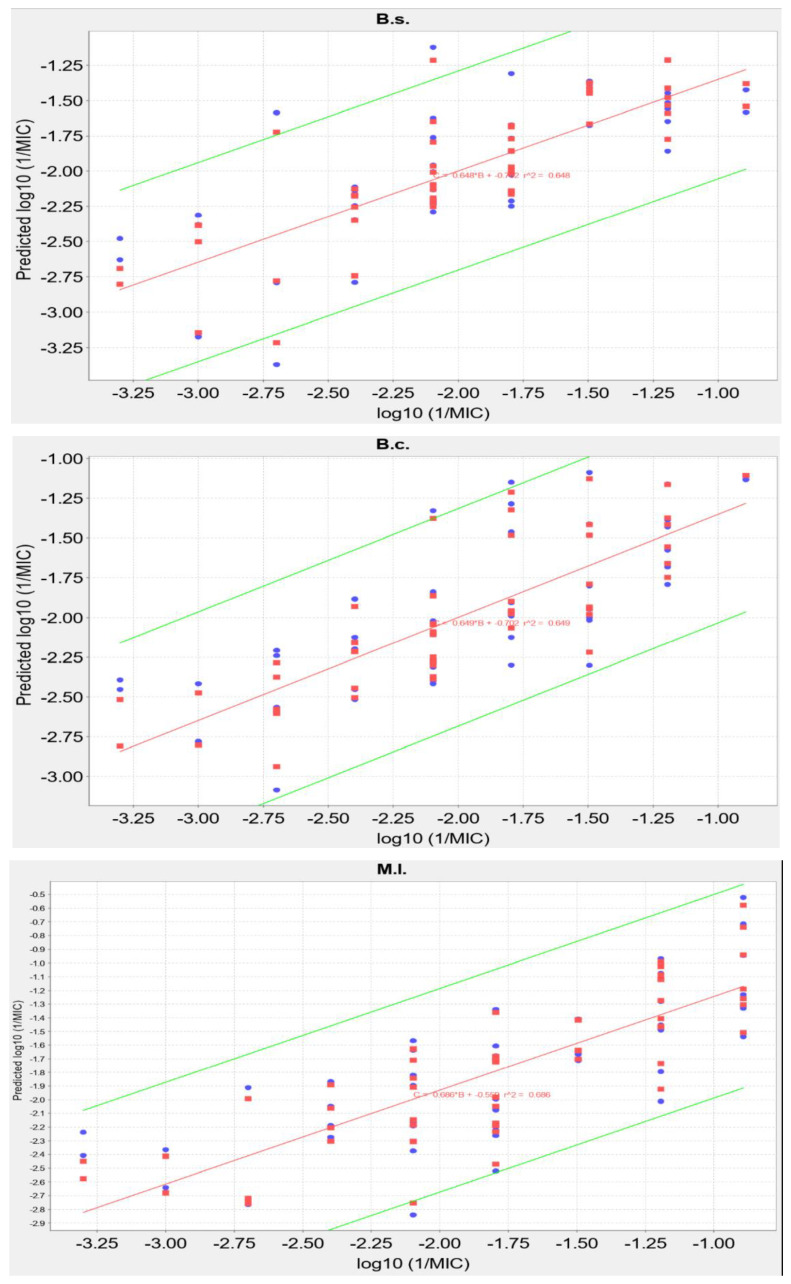
Predicted vs. experimental log_10_(1/MIC) values for the respective species for all active compounds (red squares) and leave-one-out model validation results (blue circles). Note: S. a.^*^—S. aureus ATCC 6538, S. a.^**^—S. aureus ATCC 25923, *S. e.—S. epidermidis* ATCC 12228, *B. s.—B. subtilis* ATCC 6633, *B. c.—B. cereus* ATCC 10876, *M. l.—M. luteus* ATCC 10240.

Having determined the relative stabilities of the thiosemicarbazides and *s*-triazoles, QSAR analysis was performed using SCIGRESS software (SCIGRESS v 3.4.4 www.scigress.com), to find correlations between the structure and the minimal inhibitory concentration for each microbial species separately. For these studies, only the thiosemicarbazides and *s*-triazoles that were active in the antibacterial assay were included. For the purpose of the analysis the values of the MICs were converted to Log_10_(1/MIC). The 466 descriptors used included both topological and quantum descriptors, calculated by the PM6/COSMO method (see Supplementary Materials). A fully automated procedure of descriptor selection by the enhanced replacement method (ERM) was applied to select five descriptors for best fitting of the linear regression equation (Table 4). The obtained values of R^2^ are in the range of 0.64–0.72, so, while some correlation was observed, it is not high enough for predicting unknown compounds. It is worth noticing, however, that the presence of a chlorine atom in the molecule appears to be beneficial for activity, while a bromine atom apparently decreases activity against *S. aureus* ATCC 25923. The resulting equations and fit (R^2^ and leave-one-out cross validation R^2^) are shown in Table 4. The graphs of linear regressions and cross validations are shown in Figure 4. 

### 2.6. Docking Studies

**Figure 5:**
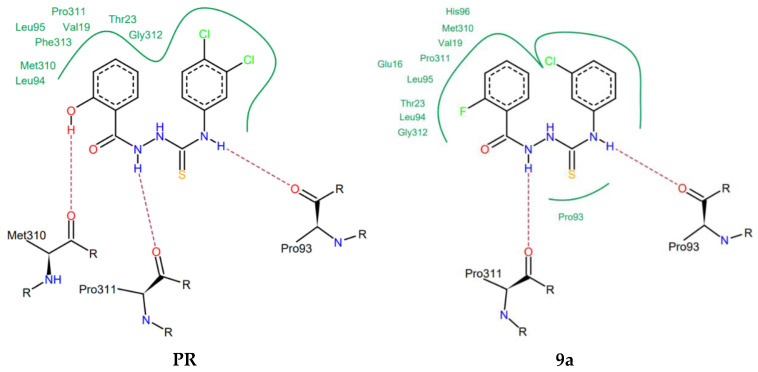

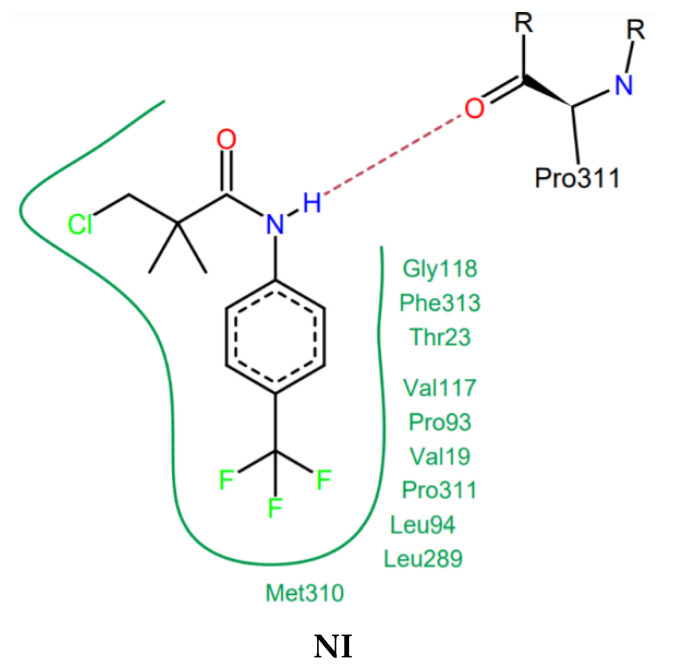
Interactions of previously reported Ddl inhibitor **PR**, our representative model thiosemicarbazide (**9a**), and native ligand **NI** in the allosteric binding site of Ddl.

As mentioned above, recently Ameryckx et al. [24,25] reported the discovery of a series of 4-aryl-1-(2-hydroxybenzoyl)thiosemicarbazides as promising allosteric inhibitors of d-alanyl-d-alanine ligase (Ddl); an essential enzyme in bacterial cell wall biosynthesis, and an important target for developing new antibacterial agents. This enzyme catalyzes the formation of d-alanile:d-alanine dipeptide, the crucial precursor of bacterial cell wall peptidoglycan, by two half-reactions. In the first half-reaction, Ddl uses one d-alanine and one ATP as substrates to produce a phosphorylated d-alanine intermediate. In the second half-reaction, Ddl uses a second d-alanine substrate to complete the reaction to the normal d-alanine:d-alanine dipeptide product [62]. According to previously reported results, the 4-(3,4-dichlorophenyl)-1-(2-hydroxybenzoyl)thiosemicarbazide (**PR**, Figure 5) was identified as one of the most potent Ddl inhibitors, with a bactericidal activity against Gram-positive bacteria, including multidrug resistant strains and, at the same time, very low cytotoxicity on a THP-1 human monocytic cell line. Although the authors concluded their extensive experiments with the judgment that the *ortho* hydroxy group is critical for the inhibitory action of benzoylthiosemicarbazides against Ddl, none of the tested compounds that were inactive on ligase exhibited an antibacterial activity at the same time. This fact supports the hypothesis that the inhibition of Ddl activity could explain, at least in part, the antibacterial effect observed for the benzoylthiosemicarbazides. 

Given the above, we decided to dock the best of our benzoylthiosemicarbazides, **9a**, **11a**, **15a**, **16a**, **9b**, **10b**, **11b**, **13b**, **15b**, and **16b,** to the allosteric site of *S. aureus* Ddl (PDB code: 2I80). The best docking scores for the thiosemicarbazides, and previously reported inhibitor **PR,** are listed in Table 5. In Figure 5 the best binding pose for representative model compound **9a** is presented. The best binding poses of the remaining thiosemicarbazides at the allosteric site of *S. aureus* Ddl are available in the Supplementary Materials. 

According to the docking results presented in Table 5, all the thiosemicarbazides are identified as binding at an allosteric site of Ddl, with much higher affinity than the native inhibitor. Their predicted binding sites overlap well with the binding site of the native inhibitor **NI,** and in all cases at least one hydrogen bond is observed between the NH group of the thiosemicarbazide skeleton and Pro311, Met 310, or Pro93 (see Supporting Material). The binding of the thiosemicarbazides **9a**, **11a**, **15a**, **16a**, **9b**, **10b**, **11b**, **13b**, **15b**, and **16b** is further stabilized by numerous hydrophobic interactions with surrounding residues, and most of these interactions are identical to those in the crystal structure of native amide **NI** in complex with *S. aureus* Ddl [62]. Notably, the binding mode of our thiosemicarbazides in the allosteric site of Ddl is stabilized by similar intermolecular interactions as predicted for previously discovered inhibitor **PR** (see Figure 5). Thus, the docking results indicate that our fluorobenzoylthiosemicarbazides can be at least theoretically considered as potential d-alanyl-d-alanine allosteric ligase inhibitors. Enzymatic experiments, however, are needed to confirm this assumption. 

## 3. Materials and Methods 

### 3.1. Chemistry

All commercial reactants and solvents were purchased from either Sigma-Aldrich (St. Louis, MO, USA) or Alfa Aesar (Karlsruhe, Germany), with the highest purity and used without further purification. The melting points were determined on a Fischer-Johns block (Fischer Scientific, Schwerte, Germany) and were uncorrected. Elemental analyses were determined by a AMZ-CHX elemental analyzer (PG, Gdańsk, Poland) and were within ±0.4% of the theoretical values. ^1^H-NMR spectra were recorded on a Bruker Avance (300 MHz) spectrometer (Bruker BioSpin GmbH, Rheinstetten, Germany). Physicochemical characterizations of the compounds **3a**, **6a**, **8a**, **9a**, **10a**, **12a**, **15a**, **2b**, **3b**, **8b**, **10b**, **12b**, **1c**, **3c**, **6c**, **8c**, **9c**, **10c**, **12c**, **3at**, **6at**, **8at**, **10at**, **12at**, **2bt**, **3bt**, **8bt**, **10bt**, **12bt**, **1ct**, **3ct**, **4ct**, **6ct**, **8ct**, **9ct**, **10ct**, **12ct**, were reported elsewhere [51,63,64,65,66,67,68,69,70]. These compounds were prepared in our lab for the purpose of this work, and their characteristics (melting points, ^1^H-NMR spectra) compared well with the characteristics already reported. The structures of the compounds **1a**, **2a**, **5a**, **7a**, **1b**, **4b**, **5b**, **6b**, **7b**, **9b**, **15b**, **2c**, **4c**, **5c**, **7c**, **15c**, **1at**, **2at**, **4at**, **5at**, **9at**, **1bt**, **2ct**, and **5ct** are known (CAS numbers); however, there are no references reporting their preparation and physicochemical characterization; therefore, these data have been included into the manuscript, and are presented in the Supplementary Materials file.

#### 3.1.1. General Procedure for Synthesis of the Thiosemicarbazides

A solution of appropriate fluorobenzoic hydrazide (0.01 mol) and an equimolar amount of aryl or alkyl isothiocyanate (0.01 mol) in anhydrous ethanol (25 mL) was heated under reflux for 10–40 min. After cooling, the solid formed was filtered off, dried, and crystallized from ethanol.

#### 3.1.2. General Procedure for Synthesis of the 1,2,4-Triazole-3-thiones

A solution of appropriate thiosemicarbazide (0.01 mol) in 2% NaOH (20 mL) was heated under reflux for 2 h. After cooling, the solution was acidified with 3M HCl whereupon a solid separated out. The solid formed was filtered off, dried, and crystallized from ethanol.

### 3.2. Antibacterial Screening

The antimicrobial activity of the title compounds was tested on six reference Gram-positive strains: *S. aureus* ATCC 25923, *S. aureus* ATCC 6538, *Staphylococcus epidermidis* ATCC 12228, *Bacillus subtilis* ATCC 6633, *B. cereus* ATCC 10876, *Micrococcus luteus* ATCC 10240, eight MSSA clinical isolates (MSSA 1–MSSA 8), and eight MRSA clinical isolates (MRSA 11–MRSA 18). For this purpose, microbial suspensions were prepared in sterile saline (0.85% NaCl) with an optical density of 0.5 McFarland standard; 150 × 10^6^ CFU/mL (CFU, colony-forming units). All the stock solutions of the tested compounds were dissolved in DMSO. Mueller-Hinton broth medium was used with a series of 2-fold dilutions of the tested substances in the range of final concentrations from 3.91 to 1000 µg/mL. For SAR analysis, compounds **7a** and **13a** were also tested at the concentration of 2000 µg/mL. Cefuroxime was used as positive control.

The in vitro antibacterial activity of the tested compounds was screened on the basis of MIC (minimal inhibitory concentration), defined as the lowest concentration of the compound at which there was no visible growth of the tested microorganisms. Determination of the MIC values was achieved by a broth microdilution method, according to CLSI recommendation [71]. MBC (minimal bactericidal concentration), defined as the lowest concentration of compound that resulted in a > 99.9% reduction in CFU of the initial inoculum, was determined by plating out the contents of wells (20 µL), that showed no visible growth of bacteria, onto Mueller-Hinton agar plates, and incubating at 35 °C for 18 h. Both MIC and MBC values are given in µg/mL, according to the CLSI reference [35].

#### Time-Kill Assay

The time-kill curve analyses were performed by culturing *Staphylococcus aureus* ATCC 25923 in Mueller-Hinton broth medium (bioMerieux, France), in the presence of seven antimicrobial-concentrations, in doubling dilutions ranging from 3.9 to 250 µg/mL (⅟_2_ × MIC to 8 × MIC) of compounds **15a**, **15b**, and **16b**. Subsequently, 0.5 McFarland Standard (1.5 × 10^8^ CFU/mL, colony-forming units per milliliter) was prepared in MHB medium from culture grown in MHB medium overnight at 35 °C. Next, 1 µL of inoculum was refreshed into 10 mL MHB medium in tubes with antimicrobial-free (control) and with various concentrations of compounds **15a**, **15b**, and **16b** (multiples MICs—⅟_2_ × MIC; 1 × MIC, 2 × MIC, 4 × MIC and 8 × MIC), and incubated at 35 °C and shaking at 150 rpm. Aliquots (1 µL) of the cultures were removed at 0, 2, 4, 6, 8, 16, 20, and 24 h, and serial 10-fold dilution were prepared in PBS buffer before planting on a Mueller-Hinton agar (bioMerieux, France, Grenoble) plate. For samples obtained from each time-point, the number of viable colonies was determined on a compound-free MHA plate following incubation at 24 h at 35 °C (number of colonies as reciprocal of the dilution factor). The lower limit for the colony counts was 5 to 300 CFU on the plate with proper dilution. Antimicrobial compounds were considered to the bactericidal at the lowest concentration that reduced the original inoculum by 99.9% for each of the indicated time-points [72]. 

### 3.3. Docking Studies

The docking simulations were performed using the FlexX docking module of the LeadIT environment as implemented in the LeadIT 2.1.9 program (BioSolveIT GmbH, Augustin, Germany) using the crystal structure of *Staphylococcus aureus* d-alanine:d-alanine ligase in complex with 3-chloro-2,2-dimethyl-*N*-[4-(trifluoromethyl)phenyl]propanamide (PDB code 2I80). The active sites were defined to include all of the atoms within 6.5 Å radius of the native ligand. To validate the docking protocol, ligands co-crystallized with the proteins were initially docked into the crystal structure of Ddl; the best conformations obtained were practically identical with the experimental ones. Subsequently, the studied thiosemicarbazides were docked using the same docking parameters. The first 100 top ranked docking poses were saved for each docking run.

## 4. Conclusions

We have shown that for a range of fluorobenzoylthiosemicarbazides, alteration of the electronic or structural nature of the thiosemicarbazide, by incorporation of the N4 alkyl or electron-donating aryl system or by the preparation of cyclic derivatives with the 1,2,4-triazole scaffold, offers no advantages over the N4 electron-withdrawing aryl system. The activity of the N4 electron-withdrawing aryl thiosemicarbazides is highly dependent on the steric effects of the substituents. The optimum activity lies with trifluoromethyl derivatives such as **15a**, **15b**, and **16b**. The docking results support the idea presented by Ameryckx et al. [24,25] that these compounds are able to act as allosteric inhibitors of d-alanyl-d-alanine ligase. Enzymatic experiments, however, are needed to provide evidence for this assumption.

## Figures and Tables

**Table 1:** In vitro antibacterial activities of the fluorobenzoylthiosemicarbazides of the *ortho* set **I** (**1a**–**16a**), *meta* set **II** (**1b**–**16b**), and *para* set **III** (**1c**–**16c**) expressed as minimum inhibitory concentrations (MICs) (μg/mL, [mM]).

	R^1^	R^2^	cLog*P*	S. a.^*^	S. a.^**^	*S. e.*	*B. s.*	*B. c.*	*M. l.*
***3a***	2F	Ph	2.26	500 [1.73]	500 [1.73]	500 [1.73]	250 [0.87]	250 [0.87]	250 [0.87]
***4a***	2F	1-naph	3.42	250 [0.74]	125 [0.37]	250 [0.74]	125 [0.37]	125 [0.37]	125 [0.37]
***8a***	2F	*para*-FPh	2.42	250 [0.81]	250 [0.81]	250 [0.81]	125 [0.41]	125 [0.41]	62.5 [0.20]
***9a***	2F	*meta*-ClPh	2.91	31.25 [0.10]	62.5 [0.19]	31.25 [0.10]	15.63 [0.05]	31.25 [0.10]	7.82 [0.02]
***10a***	2F	*para*-ClPh	2.94	250 [0.77]	500 [1.54]	250 [0.77]	500 [1.54]	31.25 [0.10]	7.82 [0.02]
***11a***	2F	*meta*-BrPh	3.04	62.5 [0.17]	62.5 [0.17]	31.25 [0.09]	15.63 [0.04]	15.63 [0.04]	7.82 [0.02]
***15a***	2F	*meta*-CF_3_Ph	3.13	15.63 [0.04]	15.63 [0.04]	7.82 [0.02]	7.82 [0.02]	7.82 [0.02]	7.82 [0.02]
***16a***	2F	*para*-CF_3_Ph	3.15	500 [1.40]	500 [1.40]	125 [0.35]	7.82 [0.02]	15.63 [0.04]	7.82 [0.02]
***3b***	3F	Ph	2.28	250 [0.87]	250 [0.87]	1000 [3.46]	250 [0.87]	250 [0.87]	125 [0.44]
***4b***	3F	1-naph	3.44	250 [0.74]	125 [0.37]	250 [0.74]	125 [0.37]	125 [0.37]	125 [0.37]
***5b***	3F	*meta*-tol	2.71	n.a.	125 [0.41]	n.a.	n.a.	n.a.	250 [0.83]
***6b***	3F	*para*-tol	2.73	1000 [3.30]	1000 [3.30]	1000 [3.30]	1000 [3.30]	1000 [3.30]	1000 [3.30]
***7b***	3F	*meta*-FPh	2.42	62.5 [0.20]	62.5 [0.20]	125 [0.41]	62.5 [0.20]	62.5 [0.20]	31.25 [0.10]
***8b***	3F	*para*-FPh	2.44	250 [0.81]	125 [0.41]	500 [1.62]	125 [0.41]	250 [0.81]	250 [0.81]
***9b***	3F	*meta*-ClPh	2.94	15.63 [0.05]	15.63 [0.05]	31.25 [0.10]	31.25 [0.10]	15.63 [0.05]	7.82 [0.02]
***10b***	3F	*para*-ClPh	2.96	15.63 [0.05]	7.82 [0.02]	7.82 [0.02]	15.63 [0.05]	62.5 [0.19]	7.82 [0.02]
***11b***	3F	*meta*-BrPh	3.07	31.25 [0.09]	15.63 [0.04]	62.5 [0.17]	15.63 [0.04]	15.63 [0.04]	15.63 [0.04]
***12b***	3F	*para*-BrPh	3.09	62.5 [0.17]	31.25 [0.09]	1000 [2.72]	125 [0.34]	62.5 [0.17]	15.63 [0.04]
***13b***	3F	*meta*-IPh	3.34	31.25 [0.08]	15.63 [0.04]	62.5 [0.15]	15.63 [0.04]	15.63 [0.04]	15.63 [0.04]
***15b***	3F	*meta*-CF_3_Ph	3.15	15.63 [0.04]	31.25 [0.09]	31.25 [0.09]	31.25 [0.09]	31.25 [0.09]	15.63 [0.04]
***16b***	3F	*para*-CF_3_Ph	3.18	15.63 [0.04]	15.63 [0.04]	31.25 [0.09]	31.25 [0.09]	15.63 [0.04]	15.63 [0.04]
***8c***	4F	*para*-FPh	2.47	250 [0.81]	62.5 [0.20]	250 [0.81]	125 [0.41]	125 [0.41]	62.5 [0.20]
***9c***	4F	*meta*-ClPh	2.96	250 [0.77]	n.a.	n.a.	62.5 [0.19]	62.5 [0.19]	15.63 [0.05]
***10c***	4F	*para*-ClPh	2.98	62.5 [0.19]	31.25 [0.10]	125 [0.39]	62.5 [0.19]	31.25 [0.10]	15.63 [0.05]
***11c***	4F	*meta*-BrPh	3.09	62.5 [0.17]	125 [0.34]	125 [0.34]	31.25 [0.09]	31.25 [0.09]	15.63 [0.04]
***12c***	4F	*para*-BrPh	3.12	62.5 [0.17]	31.25 [0.09]	62.5 [0.17]	31.25 [0.09]	31.25 [0.09]	15.63 [0.04]
***13c***	4F	*meta*-IPh	3.36	31.25 [0.08]	15.63 [0.04]	125 [0.30]	62.5 [0.15]	31.25 [0.08]	15.63 [0.04]
***14c***	4F	*para*-IPh	3.39	125 [0.30]	62.5 [0.15]	125 [0.30]	62.5 [0.15]	62.5 [0.15]	15.63 [0.04]
***15c***	4F	*meta*-CF_3_Ph	3.18	125 [0.35]	125 [0.35]	125 [0.35]	125 [0.35]	125 [0.35]	62.5 [0.18]
***16c***	4F	*para*-CF_3_Ph	3.20	125 [0.35]	62.5 [0.18]	125 [0.35]	62.5 [0.18]	62.5 [0.18]	31.25 [0.09]
***Cef.***				0.98 [0.002]	0.49 [0.001]	0.24 [0.0006]	15.63 [0.04]	31.25 [0.07]	0.98 [0.002]

Note: S. a.^*^—S. aureus ATCC 6538, S. a.^**^—S. aureus ATCC 25923, *S. e.—S. epidermidis* ATCC 12228, *B. s.—B. subtilis* ATCC 6633, *B. c.—B. cereus* ATCC 10876, *M. l.—M. luteus* ATCC 10240. Cef.—cefuroxime. n.a.—no active. The remaining thiosemicarbazides (**1a**, **2a**, **5a**–**7a**, **12a**–**14a**, **1b**, **2b**, **14b**, **1c**–**7c**) showed no antibacterial activity. The cLog*P* values were obtained through the online data server, Molinspiration [36].

**Table 2:** In vitro antibacterial activities of **15a**, **15b**, and **16b** against clinical isolates of methicillin-sensitive and methicillin-resistant *Staphylococcus aureus,* expressed as MICs (μg/mL, [mM]).

Strain	15a	15b	16b	Vancomycin
MSSA-1	15.63 [0.04]	31.25 [0.09]	7.82 [0.02]	0.39 [0.0003]
MSSA-2	15.63 [0.04]	31.25 [0.09]	15.63 [0.04]	0.78 [0.0005]
MSSA-3	7.82 [0.02]	15.63 [0.04]	7.82 [0.02]	0.39 [0.0003]
MSSA-4	15.63 [0.04]	31.25 [0.09]	15.63 [0.04]	0.78 [0.0005]
MSSA-5	7.82 [0.02]	15.63 [0.04]	15.63 [0.04]	0.78 [0.0005]
MSSA-6	7.82 [0.02]	15.63 [0.04]	15.63 [0.04]	0.78 [0.0005]
MSSA-7	7.82 [0.02]	15.63 [0.04]	7.82 [0.02]	0.78 [0.0005]
MSSA-8	7.82 [0.02]	15.63 [0.04]	15.63 [0.04]	1.56 [0.001]
MRSA-11	15.63 [0.04]	31.25 [0.09]	7.82 [0.02]	0.78 [0.0005]
MRSA-12	7.82 [0.02]	31.25 [0.09]	3.92 [0.01]	0.39 [0.0003]
MRSA-13	7.82 [0.02]	31.25 [0.09]	7.82 [0.02]	0.78 [0.0005]
MRSA-14	15.63 [0.04]	15.63 [0.04]	7.82 [0.02]	0.78 [0.0005]
MRSA-15	15.63 [0.04]	31.25 [0.09]	7.82 [0.02]	0.78 [0.0005]
MRSA-16	7.82 [0.02]	15.63 [0.04]	7.82 [0.02]	0.78 [0.0005]
MRSA-17	15.63 [0.04]	15.63 [0.04]	3.92 [0.01]	0.78 [0.0005]
MRSA-18	15.63 [0.04]	15.63 [0.04]	7.82 [0.02]	0.78 [0.0005]

Note: multidrug-sensitive clinical isolates of *S. aureus* (MSSA1–MSSA8) and multidrug-resistant clinical isolates of *S. aureus* (MRSA11–MRSA18) from hospital sources were obtained from wound swabs.

**Table 3:** In vitro antibacterial activities of the *s*-triazoles of *ortho* set **IV** (**1at**–**16at**), *meta* set **V** (**1bt**–**16bt**), and *para* set **VI** (**1ct**–**16ct**) expressed as MICs (μg/mL, [mM]).

	R^1^	R^2^	cLog*P*	S. a.^*^	S. a.^**^	*S. e.*	*B. s.*	*B. c.*	*M. l.*
***1at***	2F	Pr	2.56	1000 [4.21]	125 [0.53]	1000 [4.21]	1000 [4.21]	500 [2.11]	500 [2.11]
***8at***	2F	*para*-FPh	3.12	500 [1.73]	250 [0.86]	n.a.	250 [0.86]	500 [1.73]	125 [0.43]
***11at***	2F	*meta*-BrPh	3.95	500 [1.43]	125 [0.36]	125 [0.36]	125 [0.36]	500 [1.43]	500 [1.43]
***12at***	2F	*para*-BrPh	3.76	1000 [2.86]	500 [1.43]	1000 [2.86]	1000 [2.86]	1000 [2.86]	1000 [2.86]
***13at***	2F	*meta*-IPh	4.22	125 [0.31]	62.5 [0.16]	125 [0.31]	15.63 [0.04]	125 [0.31]	125 [0.31]
***14at***	2F	*para*-IPh	4.04	62.5 [0.16]	125 [0.31]	62.5 [0.16]	62.5 [0.16]	250 [0.63]	62.5 [0.16]
***15at***	2F	*meta*-CF_3_Ph	4.03	62.5 [0.18]	62.5 [0.18]	125 [0.37]	62.5 [0.18]	31.25 [0.09]	62.5 [0.18]
***16at***	2F	*para*-CF_3_Ph	3.85	250 [0.74]	62.5 [0.18]	250 [0.74]	250 [0.74]	125 [0.37]	62.5 [0.18]
***9bt***	3F	*meta*-ClPh	3.84	125 [0.41]	62.5 [0.20]	250 [0.82]	250 [0.82]	125 [0.41]	62.5 [0.20]
***10bt***	3F	*para*-ClPh	3.65	250 [0.82]	31.25 [0.10]	500 [1.64]	125 [0.41]	250 [0.82]	31.25 [0.10]
***11bt***	3F	*meta*-BrPh	3.97	62.5 [0.18]	62.5 [0.18]	125 [0.36]	125 [0.36]	62.5 [0.18]	125 [0.36]
***16bt***	3F	*para*-CF_3_Ph	3.87	250 [0.74]	125 [0.37]	500 [1.47]	250 [0.74]	250 [0.74]	125 [0.37]
***8ct***	4F	*para*-FPh	3.16	500 [1.73]	500 [1.73]	1000 [3.46]	500 [1.73]	500 [1.73]	500 [1.73]
***10ct***	4F	*para*-ClPh	3.68	125 [0.41]	31.25 [0.10]	125 [0.41]	125 [0.41]	125 [0.41]	125 [0.41]
***12ct***	4F	*para*-BrPh	3.81	125 [0.36]	62.5 [0.18]	125 [0.36]	62.5 [0.18]	125 [0.36]	62.5 [0.18]
***13ct***	4F	*meta*-IPh	4.27	125 [0.31]	62.5 [0.16]	125 [0.31]	62.5 [0.16]	62.5 [0.16]	62.5 [0.16]
***15ct***	4F	*meta*-CF_3_Ph	4.08	250	125 [0.37]	250 [0.74]	125 [0.37]	125 [0.37]	62.5 [0.18]
***16ct***	4F	*para*-CF_3_Ph	3.90	n.a.	n.a.	n.a.	n.a.	125 [0.37]	62.5 [0.18]
***Cef.***				0.98 [0.002]	0.49 [0.001]	0.24 [0.0006]	15.63 [0.04]	31.25 [0.07]	0.98 [0.002]

Note: S. a.^*^—S. aureus ATCC 6538, S. a.^**^—S. aureus ATCC 25923, *S. e.—S. epidermidis* ATCC 12228, *B. s.—B. subtilis* ATCC 6633, *B. c.—B. cereus* ATCC 10876, *M. l.—M. luteus* ATCC 10240. Cef.—cefuroxime. n.a.—no active. Remaining triazoles (**2at**–**7at**, **9at**, **10at**, **1bt**–**8bt**, **12bt**-**15bt**, **1ct**–**7ct**, **9ct**, **11ct**, **14ct**) showed no antibacterial activity. The cLog*P* values were obtained through the online data server, Molinspiration [36].

**Table 4:** Parameters of the five descriptor multiple linear regression QSAR models of antibacterial activity.

Species	R^2^	CV r^2^	Equation
S. a.*	0.7229	0.6346	Log_10_(1/MIC) = 0.4570 × Chlorine count + 5851.7193 × highest electrophilic susceptibility on O − 275.6544 × ring count all aromatic/MW − 1600.7357 × highest nucleophilic susceptibility/MW − 0.7411 × ln(highest nucleophilic susceptibility on H) − 3.6088
S. a.**	0.6376	0.5042	Log_10_(1/MIC) = −0.8910 × Bromine count + 68.6203 × highest electrophilic susceptibility on C − 10.8611 × highest radical susceptibility on C + 3544.5345 × highest electrophilic susceptibility on O + 0.0205 × hydrophobicity weighted positive area − 6.2195
*S. e*.	0.6589	0.5465	Log_10_(1/MIC) = 0.6767 × Chlorine count + 23.1042 × highest radical susceptibility on C + 0.0282 × atomic charge weighted positive area-atomic charge weighted negative area − 5541.1048 × highest nucleophilic susceptibility on C/MW − 1.7821 × ln(Csp^2^ bonded to 2 C) + 4.8737
*B. s.*	0.6477	0.5163	Log_10_(1/MIC) = 0.3005 × Chlorine count − 40.9730 × all count/MW − 5.3291 × highest nucleophilic susceptibility2 + 1.3923 × ln(rotatable bond count) + 2.26699e-03 × 1.0/highest nucleophilic susceptibility on H − 0.0468
*B. c.*	0.6493	0.5250	Log_10_(1/MIC) = 8728.8101 × highest electrophilic susceptibility on O − 16.0298 × highest radical susceptibility on C2 − 16.8597 × 1.0/log P + 1.64514e-03 × 1.0/highest nucleophilic susceptibility on H − 2.7091 × sqrt(ring count all aromatic) + 4.5796
*M. l.*	0.6858	0.5990	Log_10_(1/MIC) = 0.7892 × Chlorine count + 5623.4960 × highest electrophilic susceptibility on O − 344.9316 × ring count all aromatic/MW − 1613.2362 × highest nucleophilic susceptibility/MW + 1.84660e-03 × 1.0/highest radical susceptibility on H + 0.3114

Note: *S. a*.^*^—*S. aureus* ATCC 6538, *S. a*.^**^—*S. aureus* ATCC 25923, *S. e*.—*S. epidermidis* ATCC 12228, *B. s.*—*B. subtilis* ATCC 6633, *B. c*.—*B. cereus* ATCC 10876, *M. l*.—*M. luteus* ATCC 10240.

**Table 5:** FleXx docking scores of the thiosemicarbazides **9a**, **11a**, **15a**, **16a**, **9b**, **10b**, **11b**, **13b**, **15b**, and **16b** in relation to *S. aureus* d-alanyl-d-alanine ligase (Ddl).

9a	11a	15a	16a	9b	10b	11b	13b	15b	16b	PR	NI
−25.9	−23.4	−28.4	−25.4	−27.9	−28.3	−28.5	−26.0	−27.9	−28.2	−27.7	−14.3

Note: **PR**—4-(3,4-dichlorophenyl)-1-(2-hydroxybenzoyl)thiosemicarbazide; previously reported Ddl inhibitor [24]; **NI**—native inhibitor.

## Data Availability

Not applicable.

## Supplementary Materials

The following are available online: steric and electronic parameters for F, Cl, Br, I, and CF_3_ substituents, physicochemical characterization of the thiosemicarbazides and *s*-triazoles, list of descriptors used in QSAR modeling, docking binding poses obtained for all studied compounds.

## Sample Availability

Samples of title compounds are available from the authors.
