# Supplementary material for: Antibacterial Activity of Fluorobenzoylthiosemicarbazides and Their Cyclic Analogues with 1,2,4-Triazole Scaffold
Source: Molecules. 2020 Dec 31;26(1):170. doi: 10.3390/molecules26010170 (PMC7796209; doi:10.3390/molecules26010170)
Supplement: Supplementary file 1 [file molecules-26-00170-s001.pdf]

molecules

Supplementary Material

Antibacterial activity of fluorobenzoylthiosemicarbazides and their cyclic analogues with 1,2,4-triazole scaffold

Urszula Kosikowska [1], Monika Wujec [2], Nazar Trotsko [2], Wojciech Płonka [3], Piotr Paneth [4] and Agata Paneth [2,*]

[1] Department of Pharmaceutical Microbiology, Faculty of Pharmacy, Medical University of Lublin, Chodźki 1, 20-093 Lublin, Poland; urszula.kosikowska@umlub.pl
[2] Department of Organic Chemistry, Faculty of Pharmacy, Medical University of Lublin, Chodźki 4a, 20-093 Lublin, Poland; monika.wujec@umlub.pl, nazar.trotsko@umlub.pl, agata.paneth@umlub.pl
[3] FQS-Fujitsu Poland, Parkowa 11, 33-332 Kraków, Poland; w.plonka@fqs.pl
[4] Institute of Applied Radiation Chemistry, Faculty of Chemistry, Lodz University of Technology, Żeromskiego 116, 90-924 Lodz, Poland; piotr.paneth@p.lodz.pl

*correspondence: agata.paneth@umlub.pl (A.P.)

Table of contents:

1. **Table S1.** Steric and electronic parameters for F, Cl, Br, I, and CF_3 substituents
2. Physicochemical characterisation of the thiosemicarbazides
3. Physicochemical characterization of the 1,2,4-triazole-3-thiones
4. List of descriptors used in QSAR modeling
5. Docking binding poses

Table S1. Steric and electronic parameters for F, Cl, Br, I, and CF_3 substituents

	σ_m	σ_I	MR	Es	σ_v	π
**F**	0.34	0.52	5.02	-2.40	0.27	0.14
**Cl**	0.37	0.47	0.92	-0.46	0.55	0.71
**Br**	0.39	0.50	6.03	-0.97	0.65	0.86
**I**	0.35	0.39	8.88	-1.16	0.78	1.12
**CF_3**	0.43	0.42	13.94	-1.40	0.91	0.88

Note: σ_m – Hammett substituent constant; σ_I – inductive substituent constant; *MR* – molar refractivity parameter, *Es* – Taft size parameter; σ_v – Charton's steric parameter, π – Hansch substituent constant

Physicochemical characterisation of the thiosemicarbazides

1-(2-fluorobenzoyl)-4-propylthiosemicarbazide (**1a**). CAS number: 901345-11-7. Yield: 45%. M.p. 140-142°C. ^{1}H-NMR (DMSO-d6) δ (ppm): 0.90 (t, 3H, CH_3, *J* = 9.0 Hz); 1.58 (sext, 2H, CH_2, *J* = 9.0Hz); 3.39 (q, 2H, CH_2, *J* = 9.0Hz); 7.32-7.41 (m, 2H, ArH); 7.60-7.69 (m, 1H, ArH); 7.86 (dt, 1H, ArH, *J1* = 1.8 Hz, *J2* = 6.0 Hz); 8.00 (s, 1H, NH); 9.43 (s, 1H, NH); 10.18 (s, 1H, NH). Anal. calcd. for $C_{11}H_{14}FN_3OS$: C, 51.75; H, 5.53; N, 16.46. Found: C, 51.88; H, 5.49; N, 16.76.

4-butyl-1-(2-fluorobenzoyl)thiosemicarbazide (**2a**). CAS number: 443634-89-7. Yield: 40%. M.p. 145-147°C. ^{1}H-NMR (DMSO-d6) δ (ppm): 0.95 (quint, 3H, CH_3, *J* = 9 Hz); 1.33 (sext, 2H, CH_2, *J* = 9 Hz); 1.55 (quint, 2H, CH_2, *J* = 9 Hz); 3.49 (q, 2H, CH_2, *J* = 9 Hz); 7.34-7.89 (m, 4H, ArH); 7.97 (s, 1H, NH); 9.42 (s, 1H, NH); 10.17 (s, 1H, NH). Anal. calcd. for $C_{12}H_{16}FN_3OS$: C, 53.51; H, 5.99; N, 15.60. Found: C, 53.55; H, 5.78; N, 15.62.

1-(2-fluorobenzoyl)-4-(1-naphthyl)thiosemicarbazide (**4a**). CAS number: 891549-13-6. Yield: 64%. M.p. 154-156°C. ^{1}H-NMR (DMSO-d6) δ (ppm): 7.35-8.03 (m, 11H, ArH); 9.95 (s, 1H, NH); 10.03 (s, 1H, NH); 10.54 (s, 1H, NH). Anal. calcd. for $C_{18}H_{14}FN_3OS$: C, 63.70; H, 4.16; N, 12.38. Found: C, 63.75; H, 4.22; N, 12.21.

1-(2-fluorobenzoyl)-4-(3-tolyl)thiosemicarbazide (**5a**). CAS number: 443296-15-9. Yield: 82%. M.p. 168-170°C. ^{1}H-NMR (DMSO-d6) δ (ppm): 2.57 (s, 3H, CH_3); 7.03-7.94 (m, 8H, ArH); 9.77 (s, 1H, NH); 9.85 (s, 1H, NH); 10.38 (s, 1H, NH). Anal. calcd. for $C_{15}H_{14}FN_3OS$: C, 59.39; H, 4.65; N, 13.85. Found: C, 59.57; H, 4.60; N, 13.76.

1-(2-fluorobenzoyl)-4-(3-fluorophenyl)thiosemicarbazide (**7a**). CAS number: 894237-30-0. Yield: 67%. M.p. 172-174°C. ^{1}H-NMR (DMSO-d6) δ (ppm): 7.17-7.92 (m, 8H, ArH); 9.85 (s, 1H, NH); 10.01 (s, 1H, NH); 10,40 (s, 1H, NH). Anal. calcd. for $C_{14}H_{11}F_2N_3OS$: C, 54.71; H, 3.61; N, 13.67. Found: C, 54.59; H, 3.68; N, 13.77.

4-(3-bromophenyl)-1-(2-fluorobenzoyl)thiosemicarbazide (**11a**). Yield: 79%. M.p. 166-168°C. ^{1}H-NMR (DMSO-d6) δ (ppm): 7.26-7.86 (m, 8H, ArH); 9.81 (s, 1H, NH); 9.98 (s, 1H, NH); 10.33 (s, 1H, NH). Anal. calcd. for $C_{14}H_{11}BrFN_3OS$: C, 45.66; H, 3.01; N, 11.41. Found: C, 45.88; H, 3.09; N, 11.26.

1-(2-fluorobenzoyl)-4-(3-iodophenyl)thiosemicarbazide (**13a**). Yield: 83%. M.p. 176-178°C. ^{1}H-NMR (DMSO-d6) δ (ppm): 7.17-7.92 (m, 8H, ArH); 9.84 (s, 1H, NH); 10.02 (s, 1H, NH); 10,42 (s, 1H, NH). Anal. calcd. for $C_{14}H_{11}FIN_3OS$: C, 40.49; H, 2.67; N, 10.12. Found: C, 40.20; H, 3.01; N, 10.29.

1-(2-fluorobenzoyl)-4-(4-iodophenyl)thiosemicarbazide (**14a**). Yield: 89%. M.p. 178-180°C. ^{1}H-NMR (DMSO-d6) δ (ppm): 7.21-7.91 (m, 8H, ArH); 9.84 (s, 1H, NH); 9.98 (s, 1H, NH); 10.39 (s, 1H, NH). Anal. $C_{14}H_{11}FIN_3OS$ (C, H, N). Anal. calcd. for $C_{14}H_{11}FIN_3OS$: C, 40.49; H, 2.67; N, 10.12. Found: C, 40.51; H, 2.56; N, 10.20.

1-(2-fluorobenzoyl)-4-(4-trifluoromethylphenyl)thiosemicarbazide (**16a**). Yield: 88%. M.p. 198-200°C. ^{1}H-NMR (DMSO-d6) δ (ppm): 7.37-7.90 (m, 8H, ArH); 10.15 (m, 2H, NH); 10.47 (s, 1H, NH). Anal. Anal. calcd. for $C_{15}H_{11}F_4N_3OS$: C, 50.42; H, 3.10; N, 11.76. Found: C, 50.53; H, 3.33; N, 11.54.

1-(3-fluorobenzoyl)-4-propylthiosemicarbazide (**1b**). CAS number: 891047-47-5. Yield: 64%. M.p. 185-187°C. ^{1}H-NMR (DMSO-d6) δ (ppm): 0.88 (t, 3H, CH_3, *J* = 9 Hz); 1.55-1.58 (m, 2H, CH_2); 3.38-3.47 (m, 2H, CH_2); 7.49-7.83 (m, 4H, ArH); 8.20 (s, 1H, NH); 9.33 (s, 1H, NH); 10.46 (s, 1H, NH). Anal. $C_{18}H_{14}FN_3OS$ (C, H, N). Anal. calcd. for $C_{11}H_{14}FN_3OS$: C, 51.75; H, 5.53; N, 16.46. Found: C, 51.80; H, 5.57; N, 16.77.

1-(3-fluorobenzoyl)-4-(1-naphthyl)thiosemicarbazide (**4b**). CAS number: 891548-57-5. Yield: 90%. M.p. 170-172°C. ^{1}H-NMR (DMSO-d6) δ (ppm): 7.55-8.02 (m, 11H, ArH); 9.90 (s, 1H, NH); 10.12 (s, 1H, NH); 10.18 (s, 1H, NH); 10.86 (s, 1H, NH). Anal. $C_{18}H_{14}FN_3OS$ (C, H, N). Anal. calcd. for $C_{18}H_{14}FN_3OS$: C, 63.70; H, 4.16; N, 12.38. Found: C, 63.58; H, 4.25; N, 12.23.

1-(3-fluorobenzoyl)-4-(3-tolyl)thiosemicarbazide (**5b**). CAS number: 894216-38-7. Yield: 89%. M.p. 139-141°C. ^{1}H-NMR (DMSO-d6) δ (ppm): 2.29 (s, 3H, CH3); 6.97-7.81 (m, 8H, ArH); 9.73 (s, 1H, NH); 9.79 (s, 1H, NH); 10.64 (s, 1H, NH). Anal. calcd. for $C_{15}H_{14}FN_3OS$: C, 59.39; H, 4.65; N, 13.85. Found: C, 59.46; H, 4.49; N, 13.77.

1-(3-fluorobenzoyl)-4-(4-tolyl)thiosemicarbazide (**6b**). CAS number: 894216-33-2. Yield: 90%. M.p. 176-158°C. ^{1}H-NMR (DMSO-d6) δ (ppm): 2.35 (s, 3H, CH3); 7.18-7.88 (m, 8H, ArH); 9.77 (s, 1H, NH); 9.84 (s, 1H, NH); 10.69 (s, 1H, NH). Anal. $C_{15}H_{14}FN_3OS$ (C, H, N). Anal. calcd. for $C_{15}H_{14}FN_3OS$: C, 59.39; H, 4.65; N, 13.85. Found: C, 59.28; H, 4.52; N, 13.72.

1,4-di(3-fluorobenzoyl)thiosemicarbazide (**7b**). CAS number: 894237-23-1. Yield: 71%. M.p. 184-186°C. ^{1}H-NMR (DMSO-d6) δ (ppm): 6.81-7.84 (m, 8H, ArH); 9.97 (s, 2H, 2NH); 10.74 (s, 1H, NH). Anal. calcd. for $C_{14}H_{11}F_2N_3OS$: C, 54.71; H, 3.61; N, 13.67. Found: C, 54.89; H, 3.51; N, 13.46.

4-(3-chlorophenyl)-1-(3-fluorobenzoyl)thiosemicarbazide (**9b**). CAS number: 894227-15-7. Yield: 77%. M.p. 175-177°C. ^{1}H-NMR (DMSO-d6) δ (ppm): 7.29-7.88 (m, 8H, ArH); 9.99 (s, 2H, 2NH); 10.75 (s, 1H, NH). Anal. calcd. for $C_{14}H_{11}ClFN_3OS$: C, 51.93; H, 3.42; N, 12.98. Found: C, 52.03; H, 3.39; N, 12.74.

4-(3-bromophenyl)-1-(3-fluorobenzoyl)thiosemicarbazide (**11b**). Yield: 71%. M.p. 183-185°C. ^{1}H-NMR (DMSO-d6) δ (ppm): 7.35-7.87 (m, 8H, ArH); 10.00 (s, 2H, 2NH); 10.75 (s, 1H, NH). Anal. $C_{14}H_{11}BrFN_3OS$ (C, H, N). Anal. calcd. for $C_{14}H_{11}BrFN_3OS$: C, 45.66; H, 3.01; N, 11.41. Found: C, 45.39; H, 3.21; N, 11.68.

1-(3-fluorobenzoyl)-4-(3-iodophenyl)-thiosemicarbazide (**13b**). Yield: 75%. M.p. 202-204°C. ^{1}H-NMR (DMSO-d6) δ (ppm): 7.01-7.89 (m, 8H, ArH); 9.70 (s, 1H, NH); 9.86 (s, 1H, NH); 10.73 (s, 1H, NH). Anal. $C_{14}H_{11}FIN_3OS$ (C, H, N). Anal. calcd. for $C_{14}H_{11}FIN_3OS$: C, 40.49; H, 2.67; N, 10.12. Found: C, 40.26; H, 2.81; N, 10.20.

1-(3-fluorobenzoyl)-4-(4-iodophenyl)-thiosemicarbazide (**14b**). Yield: 88%. M.p. 194-196°C. ^{1}H-NMR (DMSO-d6) δ (ppm): 7.16-7.87 (m, 8H, ArH); 9.91 (s, 2H, 2NH); 10.71 (s, 1H, NH). Anal. $C_{14}H_{11}FIN_3OS$ (C, H, N). Anal. calcd. for $C_{14}H_{11}FIN_3OS$: C, 40.49; H, 2.67; N, 10.12. Found: C, 40.36; H, 2.89; N, 9.99.

1-(3-fluorobenzoyl)-4-(3-trifluoromethylphenyl)thiosemicarbazide (**15b**). Yield: 71%. M.p. 165-167°C. ^{1}H-NMR (DMSO-d6) δ (ppm): 7.54-7.90 (m, 8H, ArH); 10.06 (s, 2H, NH); 10.77(s, 1H, NH). Anal. calcd. for $C_{15}H_{11}F_4N_3OS$: C, 50.42; H, 3.10; N, 11.76. Found: C, 50.37; H, 3.22; N, 11.59.

1-(3-fluorobenzoyl)-4-(4-trifluoromethylphenyl)thiosemicarbazide (**16b**). Yield: 73%. M.p. 249-251°C. ^{1}H-NMR (DMSO-d6) δ (ppm): 7.51-7.87 (m, 8H, ArH); 10.07 (s, 2H, NH); 10.77 (s, 1H, NH). Anal. calcd. for $C_{15}H_{11}F_4N_3OS$: C, 50.42; H, 3.10; N, 11.76. Found: C, 50.55; H, 3.39; N, 11.78.

4-butyl-1-(4-fluorobenzoyl)thiosemicarbazide (**2c**). CAS number: 443296-09-1. Yield: 66%. M.p. 184-186°C. ^{1}H-NMR (DMSO-d6) δ (ppm): 0.88 (t, 3H, CH_3, J = 7.5 Hz); 1.25 (sext, 2H, CH_2, J = 7.2 Hz); 1.46 (q, 2H, CH_2, J = 7.5 Hz); 3.43 (kw, 2H, CH_2, J = 6.6 Hz); 7.34 (t, 2H, ArH, J = 9.0Hz); 7.99 (kw, 2H, ArH, J = 5.7 Hz); 8.11 (s, 1H, NH); 9.25 (s, 1H, NH); 10.34 (s, 1H, NH). Anal. calcd. for $C_{12}H_{16}FN_3OS$: C, 53.51; H, 5.99; N, 15.60. Found: C, 53.49; H, 5.81; N, 15.66.

1-(4-fluorobenzoyl)-4-(1-naphthyl)thiosemicarbazide (**4c**). CAS number: 891549-29-4. Yield: 67%. M.p. 177-179°C. ^{1}H-NMR (DMSO-d6) δ (ppm): 7.37-7.59 (m, 6H, ArH); 7.88-7.16 (m, 5H, ArH); 9.91 (s, 1H, NH); 10.15 (s, 1H, NH); 10.82 (s, 1H, NH). Anal. $C_{18}H_{14}FN_3OS$ (C, H, N). Anal. calcd. for $C_{18}H_{14}FN_3OS$: C, 63.70; H, 4.16; N, 12.38. Found: C, 63.69; H, 4.25; N, 12.42.

1-(4-fluorobenzoyl)-4-(3-tolyl)thiosemicarbazide (**5c**). CAS number: 443666-96-4. Yield: 73%. M.p. 178-180°C. ^{1}H-NMR (DMSO-d6) δ (ppm): 2.25 (s, 3H, CH3); 7.02-7.03 (m, 2H, ArH); 7.11-7.16 (m, 4H, ArH); 7.89-7.93 (m, 2H, ArH); 9.63 (s, 1H, NH); 9.72 (s, 1H, NH); 10.51 (s, 1H, NH). Anal. $C_{15}H_{14}FN_3OS$ (C, H, N). Anal. calcd. for $C_{15}H_{14}FN_3OS$: C, 59.39; H, 4.65; N, 13.85. Found: C, 59.22; H, 4.77; N, 13.91.

1-(4-fluorobenzoyl)-4-(3-fluorophenyl)thiosemicarbazide (**7c**). CAS number: 894237-00-4. Yield: 81%. M.p. 193-195°C. ^{1}H-NMR (DMSO-d6) δ (ppm): 6.99 (t, 1H, ArH, *J* = 7,5 Hz); 7.28-7.48 (m, 5H, ArH); 8.03 (kw, 2H, ArH, *J* = 5,4 Hz); 9.89 (s, 2H, 2NH,); 10.61 (s, 1H, NH). Anal. calcd. for $C_{14}H_{11}F_2N_3OS$: C, 54.71; H, 3.61; N, 13.67. Found: C, 54.70; H, 3.32; N, 13.79.

4-(3-bromophenyl)-1-(4-fluorobenzoyl)thiosemicarbazide (**11c**). Yield: 81%. M.p. 185-187°C. ^{1}H-NMR (DMSO-d6) δ (ppm): 7.27-7.39 (m, 4H, ArH); 7.50-7.54 (m, 1H, ArH); 7.72 (s, 1H, ArH); 8.02 (kw, 2H, ArH, *J* = 5.7); 9.90 (s, 2H, 2 NH); 10.61 (s, 1H, NH). Anal. $C_{14}H_{11}BrFN_3OS$ (C, H, N). Anal. calcd. for $C_{14}H_{11}BrFN_3OS$: C, 45.66; H, 3.01; N, 11.41. Found: C, 45.52; H, 3.13; N, 11.55.

1-(4-fluorobenzoyl)-4-(3-iodophenyl)thiosemicarbazide (**13c**). Yield: 79%. M.p. 193-195°C. ^{1}H-NMR (DMSO-d6) δ (ppm): 7.18-7.21 (m, 1H, ArH); 7.41-7.44 (m, 2H, ArH); 7.57-7.61 (m, 2H, ArH); 7.87 (s, 1H, ArH); 8.07-8.09 (m, 2H, ArH); 9.93 (s, 2H, 2NH); 10.66 (s, 1H, NH). Anal. calcd. for $C_{14}H_{11}FIN_3OS$: C, 40.49; H, 2.67; N, 10.12. Found: C, 40.18; H, 2.42; N, 10.33.

1-(4-fluorobenzoyl)-4-(4-iodophenyl)thiosemicarbazide (**14c**). Yield: 90%. M.p. 222-224°C. ^{1}H-NMR (DMSO-d6) δ (ppm): 7.29-7.38 (m, 4H, ArH); 7.67-7.68 (m, 2H, ArH); 8.01-8.03 (m, 2H, ArH); 9.83 (s, 2H, 2NH); 10.59 (s, 1H, NH). Anal. calcd. for $C_{14}H_{11}FIN_3OS$: C, 40.49; H, 2.67; N, 10.12. Found: C, 40.71; H, 2.93; N, 10.09.

1-(4-fluorobenzoyl)-4-(3-trifluoromethylphenyl)thiosemicarbazide (**15c**). CAS number: 905262-01-3. Yield: 85%. M.p. 208-210°C. ^{1}H-NMR (DMSO-d6) δ (ppm): 7.37 (t, 2H, ArH, *J* = 9.0Hz); 7.51-7.60 (m, 2H, ArH); 7.84 (d, 2H, ArH, *J* = 7.2 Hz); 8.04 (kw, 2H, ArH, *J* = 5.4 Hz); 10.00 (s, 2H, 2NH); 10.66 (s, 1H, NH). Anal. $C_{15}H_{11}F_4N_3OS$ (C, H, N). Anal. calcd. for $C_{15}H_{11}F_4N_3OS$: C, 50.42; H, 3.10; N, 11.76. Found: C, 50.29; H, 3.04; N, 11.50.

1-(4-fluorobenzoyl)-4-(4-trifluoromethylphenyl)thiosemicarbazide (**16c**). Yield: 91%. M.p. 222-224°C. ^{1}H-NMR (DMSO-d6) δ (ppm): 7.37 (t, 2H, ArH, *J* = 9.0Hz); 7.68-7.76 (m, 4H, ArH); 8.04 (kw, 2H, ArH, *J* = 5.7 Hz); 10.00 (s, 2H, 2NH); 10.67 (s, 1H, NH). Anal. calcd. for $C_{15}H_{11}F_4N_3OS$: C, 50.42; H, 3.10; N, 11.76. Found: C, 50.51; H, 2.97; N, 11.45.

Physicochemical characterization of the 1,2,4-triazole-3-thiones

5-(2-fluorophenyl)-4-propyl-2,4-dihydro-3*H*-1,2,4-triazole-3-thione (**1at**). CAS number: 1553509-98-0. Yield: 48%. M.p. 127-130°C. ^{1}H-NMR (DMSO-d6) δ (ppm): 0.63-0.71 (m, 3H, CH3); 1.44-1.55 (m, 2H, CH2); 3.79-3.88 (m, 2H, CH2); 7.40-7.71 (m, 4H, ArH); 14.11 (s, 1H, NH). Anal. calcd. for $C_{11}H_{12}FN_3S$: C, 55.68; H, 5.10; N, 17.71. Found: C, 55.52; H, 5.33; N, 17.49.

4-butyl-5-(2-fluorophenyl)-2,4-dihydro-3*H*-1,2,4-triazole-3-thione (**2at**). CAS number: 892217-67-3. Yield: 51%. M.p. >300°C. ^{1}H-NMR (DMSO-d6) δ (ppm): 0.69 (t, 3H, CH_3, *J* = 7.5 Hz); 1.08 (sext, 2H, CH2, *J* = 7.5 Hz); 1.44 (quint, 2H, CH2, *J* = 7.5 Hz); 14.11 (s, 1H, NH). Anal. calcd. for $C_{12}H_{14}FN_3S$: C, 57.35; H, 5.61; N, 16.72. Found: C, 57.33; H, 5.39; N, 16.90.

4-(1-naphthyl)-5-(2-fluorophenyl)-2,4-dihydro-3*H*-1,2,4-triazole-3-thione (**4at**). CAS number: 496787-38-3. Yield: 78%. M.p. 242-244°C. ^{1}H-NMR (DMSO-d6) δ (ppm): 7.07-7.14 (m, 2H, ArH); 7.40-7.45 (m, 3H, ArH); 7.54-7.58 (m, 4H, ArH); 7.99-8.02 (m, 2H, ArH); 14.44 (s, 1H, NH). Anal. calcd. for $C_{18}H_{12}FN_3S$: C, 67.27; H, 3.76; N, 13.08. Found: C, 66.98; H, 3.32; N, 12.96.

5-(2-fluorophenyl)-4-(3-tolyl)-2,4-dihydro-3*H*-1,2,4-triazole-3-thione (**5at**). CAS number: 694478-51-8. Yield: 90%. M.p. 214-216°C. ^{1}H-NMR (DMSO-d6) δ (ppm): 2.25 (s, 3H, CH3); 7.04-7.57 (m, 8H, ArH); 14.29 (s, 1H, NH). Anal. calcd. for $C_{15}H_{12}FN_3S$: C, 63.14; H, 4.24; N, 14.73. Found: C, 63.03; H, 4.41; N, 14.56.

5-(2-fluorophenyl)-4-(3-fluorophenyl)-2,4-dihydro-3*H*-1,2,4-triazole-3-thione (**7at**). Yield: 78%. M.p. 202-204°C. ^{1}H-NMR (DMSO-d6) δ (ppm): 7.27-7.56 (m, 8H, ArH); 14.32 (s, 1H, NH). Anal. calcd. for $C_{14}H_9F_2N_3S$: C, 58.12; H, 3.14; N, 14.53. Found: C, 57.91; H, 3.18; N, 14.67.

4-(3-chlorophenyl)-5-(2-fluorophenyl)-2,4-dihydro-3*H*-1,2,4-triazole-3-thione (**9at**). CAS number: 694473-88-6. Yield: 75%. M.p. 215-217°C. ^{1}H-NMR (DMSO-d6) δ (ppm): 7.25-7.38 (m, 4H, ArH); 7.52-7.61 (m, 4H, ArH); 14.31 (s, 1H, NH). Anal. calcd. for $C_{14}H_9ClFN_3S$: C, 55.00; H, 2.97; N, 13.74. Found: C, 55.03; H, 3.13; N, 13.56.

4-(3-bromophenyl)-5-(2-fluorophenyl)-2,4-dihydro-3*H*-1,2,4-triazole-3-thione (**11at**). Yield: 68%. M.p. >300°C. ^{1}H-NMR (DMSO-d6) δ (ppm): 7.13-7.65 (m, 8H, ArH); 14.28 (s, 1H, NH). Anal. calcd. for $C_{14}H_9BrFN_3S$: C, 48.01; H, 2.59; N, 12.00. Found: C, 47.99; H, 2.22; N, 12.09.

4-(3-iodophenyl)-5-(2-fluorophenyl)-2,4-dihydro-3*H*-1,2,4-triazole-3-thione (**13at**). Yield: 65%. M.p. 218-220°C. ^{1}H-NMR (DMSO-d6) δ (ppm): 7.21-7.32 (m, 4H, ArH); 7.64-7.57 (m, 2H, ArH); 7.73-7.78 (m, 2H, ArH); 14.36 (s, 1H, NH). Anal. calcd. for $C_{14}H_9FIN_3S$: C, 42.33; H, 2.28; N, 10.58. Found: C, 42.38; H, 2.53; N, 10.50.

4-(4-iodophenyl)-5-(2-fluorophenyl)-2,4-dihydro-3*H*-1,2,4-triazole-3-thione (**14at**). Yield: 88%. M.p. 284-286°C. ^{1}H-NMR (DMSO-d6) δ (ppm): 7.05-7.30 (m, 4H, ArH); 7.48-7.55 (m, 2H, ArH); 7.73-7.81 (m, 2H, ArH); 14.31 (s, 1H, NH). Anal. calcd. for $C_{14}H_9FIN_3S$: C, 42.33; H, 2.28; N, 10.58. Found: C, 42.50; H, 2.51; N, 10.36.

4-(3-trifluoromethylphenyl)-5-(2-fluorophenyl)-2,4-dihydro-3*H*-1,2,4-triazole-3-thione (**15at**). Yield: 65%. M.p. 212-214°C. ^{1}H-NMR (DMSO-d6) δ (ppm): 7.21-7.60 (m, 8H, ArH); 14.36 (s, 1H, NH). Anal. calcd. for $C_{15}H_9F_4N_3S$: C, 53.10; H, 2.67; N, 12.38. Found: C, 53.24; H, 2.45; N, 12.35.

4-(4-trifluoromethylphenyl)-5-(2-fluorophenyl)-2,4-dihydro-3*H*-1,2,4-triazole-3-thione (**16at**). Yield: 68%. M.p. 124-126°C. ^{1}H-NMR (DMSO-d6) δ (ppm): 7.19-7.96 (m, 8H, ArH); 14.39 (s, 1H, NH). Anal. calcd. for $C_{15}H_9F_4N_3S$: C, 53.10; H, 2.67; N, 12.38. Found: C, 52.93; H, 2.50; N, 12.76.

5-(3-fluorophenyl)-4-propyl-2,4-dihydro-3*H*-1,2,4-triazole-3-thione (**1bt**). CAS number: 1549486-52-3. Yield: 65%. M.p. 126-128°C. ^{1}H-NMR (DMSO-d6) δ (ppm): 0.69 (t, 3H, CH_3, *J* = 7.5 Hz); 1.51 (sext, 2H, CH_2, *J* = 7.5 Hz); 4.01 (t, 2H, CH_2, *J* = 7.5 Hz); 7.43-7.69 (m, 4H, ArH); 14.04 (s, 1H, NH). Anal. calcd. for $C_{11}H_{12}FN_3S$: C, 55.68; H, 5.10; N, 17.71. Found: C, 55.73; H, 5.09; N, 17.47.

5-(3-fluorophenyl)-4-(1-naphthyl)-2,4-dihydro-3*H*-1,2,4-triazole-3-thione (**4bt**). Yield: 67%. M.p. 287-289°C. ^{1}H-NMR (DMSO-d6) δ (ppm): 7.10-7.78 (m, 9H, ArH); 8.14 (dd, 2H, ArH, *J* = 4.2 Hz); 14.45 (s, 1H, NH). Anal. $C_{18}H_{12}FN_3S$ (C, H, N). Anal. calcd. for $C_{18}H_{12}FN_3S$: C, 67.27; H, 3.76; N, 13.08. Found: C, 67.35; H, 3.52; N, 13.19.

5-(3-fluorophenyl)-4-(3-tolyl)-2,4-dihydro-3*H*-1,2,4-triazole-3-thione (**5bt**). Yield: 80%. M.p. 234-236°C. ^{1}H-NMR (DMSO-d6) δ (ppm): 2.31 (s, 3H, CH_3); 7.09-7.44 (m, 8H, ArH); 14.24 (s, 1H, NH). Anal. calcd. for $C_{15}H_{12}FN_3S$: C, 63.14; H, 4.24; N, 14.73. Found: C, 62.97; H, 3.99; N, 14.50.

5-(3-fluorophenyl)-4-(4-tolyl)-2,4-dihydro-3*H*-1,2,4-triazole-3-thione (**6bt**). Yield: 81%. M.p. 239-241°C. ^{1}H-NMR (DMSO-d6) δ (ppm): 2.36 (s, 3H, CH_3); 7.11-8.19 (m, 8H, ArH); 14.22 (s, 1H, NH). Anal. calcd. for $C_{15}H_{12}FN_3S$: C, 63.14; H, 4.24; N, 14.73. Found: C, 63.22; H, 4.21; N, 14.58.

4,5-di(3-fluorophenyl)-2,4-dihydro-3*H*-1,2,4-triazole-3-thione (**7bt**). Yield: 65%. M.p. 254-256°C. ^{1}H-NMR (DMSO-d6) δ (ppm): 7.15-7.57 (m, 8H, ArH); 14.29 (s, 1H, NH). Anal. calcd. for $C_{14}H_9F_2N_3S$: C, 58.12; H, 3.14; N, 14.53. Found: C, 58.24; H, 3.26; N, 14.50.

4-(3-chlorophenyl)-5-(3-fluorophenyl)-2,4-dihydro-3*H*-1,2,4-triazole-3-thione (**9bt**). Yield: 68%. M.p. 256-258°C. ^{1}H-NMR (DMSO-d6) δ (ppm): 7.14-7.65 (m, 8H, ArH); 14.28 (s, 1H, NH). Anal. calcd. for $C_{14}H_9ClFN_3S$: C, 55.00; H, 2.97; N, 13.74. Found: C, 54.92; H, 3.11; N, 13.26.

4-(3-bromophenyl)-5-(3-fluorophenyl)-2,4-dihydro-3*H*-1,2,4-triazole-3-thione (**11bt**). Yield: 66%. M.p. 255-257°C. ^{1}H-NMR (DMSO-d6) δ (ppm): 7.14-7.77 (m, 8H, ArH); 14.29 (s, 1H, NH). Anal. calcd. for $C_{14}H_9BrFN_3S$: C, 48.01; H, 2.59; N, 12.00. Found: C, 47.99; H, 2.25; N, 12.21.

4-(3-iodophenyl)-5-(3-fluorophenyl)-2,4-dihydro-3*H*-1,2,4-triazole-3-thione (**13bt**). Yield: 71%. M.p. 251-253°C. ^{1}H-NMR (DMSO-d6) δ (ppm): 7.13-7.17 (m, 4H, ArH); 7.29-7.32 (m, 4H, ArH); 14.30 (s, 1H, NH). Anal. calcd. for $C_{14}H_9FIN_3S$: C, 42.33; H, 2.28; N, 10.58. Found: C, 42.25; H, 2.18; N, 10.71.

4-(4-iodophenyl)-5-(3-fluorophenyl)-2,4-dihydro-3*H*-1,2,4-triazole-3-thione (**14bt**). Yield: 77%. M.p. 234-236°C. ^{1}H-NMR (DMSO-d6) δ (ppm): 7.13-7.89 (m, 8H, ArH); 14.26 (s, 1H, NH). Anal. calcd. for $C_{14}H_9FIN_3S$: C, 42.33; H, 2.28; N, 10.58. Found: C, 42.42; H, 2.22; N, 10.31.

4-(3-trifluoromethylphenyl)-5-(3-fluorophenyl)-2,4-dihydro-3*H*-1,2,4-triazole-3-thione (**15bt**). Yield: 60%. M.p. 239-241°C. ^{1}H-NMR (DMSO-d6) δ (ppm): 7.14-7.93 (m, 8H, ArH); 14.30 (s, 1H, NH). Anal. calcd. for $C_{15}H_9F_4N_3S$: C, 53.10; H, 2.67; N, 12.38. Found: C, 53.26; H, 2.60; N, 12.21.

4-(4-trifluoromethylphenyl)-5-(3-fluorophenyl)-2,4-dihydro-3*H*-1,2,4-triazole-3-thione (**16bt**). Yield: 62%. M.p. 249-251°C. ^{1}H-NMR (DMSO-d6) δ (ppm): 7.10-8.06 (m, 8H, ArH); 14.32 (s, 1H, NH). Anal. calcd. for $C_{15}H_9F_4N_3S$: C, 53.10; H, 2.67; N, 12.38. Found: C, 52.99; H, 2.51; N, 12.50.

4-butyl-5-(4-fluorophenyl)-2,4-dihydro-3*H*-1,2,4-triazole-3-thione (**2ct**). CAS number: 694456-05-8. Yield: 65%. M.p. 108-110°C. ^{1}H-NMR (DMSO-d6) δ (ppm): 0.73 (t, 3H, CH_3, *J* = 7.2 Hz); 1.11 (sext, 2H, CH_2, *J* = 7.2 Hz); 1.48 (q, 2H, CH_2, *J* = 7.5 Hz); 4.01 (t, 2H, CH_2, *J* = 7.5 Hz); 7.41 (t, 2H, ArH, *J* = 9.0 Hz); 7.43 (m, 2H, ArH); 13.90 (s, 1H, NH). Anal. calcd. for $C_{12}H_{14}FN_3S$: C, 57.35; H, 5.61; N, 16.72. Found: C, 57.21; H, 5.88; N, 16.91.

5-(4-fluorophenyl)-4-(3-tolyl)-2,4-dihydro-3*H*-1,2,4-triazole-3-thione (**5ct**). CAS number: 1044824-22-1. Yield: 86%. M.p. 256-258°C. ^{1}H-NMR (DMSO-d6) δ (ppm): 2.32 (s, 3H, CH_3); 7.12-7.39 (m, 8H, ArH); 14.13 (s, 1H, NH). Anal. calcd. for $C_{15}H_{12}FN_3S$: C, 63.14; H, 4.24; N, 14.73. Found: C, 62.95; H, 4.20; N, 14.50.

5-(4-fluorophenyl)-4-(3-fluorophenyl)-2,4-dihydro-3*H*-1,2,4-triazole-3-thione (**7ct**). Yield: 79%. M.p. 194-196°C. ^{1}H-NMR (DMSO-d6) δ (ppm): 6.88-6.92 (m, 1H, ArH); 7.08-7.16 (m, 3H, ArH); 7.20-7.26 (m, 3H, ArH); 7.39-7.42 (m, 1H, ArH); 14.29 (s, 1H, NH). Anal. calcd. for $C_{14}H_9F_2N_3S$: C, 58.12; H, 3.14; N, 14.53. Found: C, 58.03; H, 3.12; N, 14.59.

4-(3-bromophenyl)-5-(4-fluorophenyl)-2,4-dihydro-3*H*-1,2,4-triazole-3-thione (**11ct**). Yield: 79%. M.p. 276-278°C. ^{1}H-NMR (DMSO-d6) δ (ppm): 7.25-7.65 (t, 2H, ArH, *J* = 9.0 Hz); 7.37-7.48 (m, 4H, ArH); 7.68-7.75 (m, 2H, ArH); 14.20 (s, 1H, NH). Anal. calcd. for $C_{14}H_9BrFN_3S$: C, 48.01; H, 2.59; N, 12.00. Found: C, 47.86; H, 2.53; N, 12.15.

4-(3-iodophenyl)-5-(4-fluorophenyl)-2,4-dihydro-3*H*-1,2,4-triazole-3-thione (**13ct**). Yield: 85%. M.p. 251-253°C. ^{1}H-NMR (DMSO-d6) δ (ppm): 7.24-7.30 (m, 4H, ArH); 7.37-7.40 (m, 2H, ArH); 7.84-7.86 (m, 2H, ArH); 14.17 (s, 1H, NH). Anal. calcd. for $C_{14}H_9FIN_3S$: C, 42.33; H, 2.28; N, 10.58. Found: C, 42.03; H, 2.01; N, 10.60.

4-(4-iodophenyl)-5-(4-fluorophenyl)-2,4-dihydro-3*H*-1,2,4-triazole-3-thione (**14ct**). Yield: 86%. M.p. 214-216°C. ^{1}H-NMR (DMSO-d6) δ (ppm): 7.24-7.32 (m, 4H, ArH); 7.43-7.46 (m, 2H, ArH); 7.92-7.93 (m, 2H, ArH); 14.25 (s, 1H, NH). Anal. calcd. for $C_{14}H_9FIN_3S$: C, 42.33; H, 2.28; N, 10.58. Found: C, 42.51; H, 2.40; N, 10.37.

4-(3-trifluoromethylphenyl)-5-(4-fluorophenyl)-2,4-dihydro-3*H*-1,2,4-triazole-3-thione (**15ct**). Yield: 77%. M.p. 283-285°C. ^{1}H-NMR (DMSO-d6) δ (ppm): 7.23-7.26 (m, 2H, ArH); 7.37-7.39 (m, 2H, ArH); 7.70-7.73 (m, 2H, ArH); 7.85-7.90 (m, 2H, ArH); 14.25 (s, 1H, NH). Anal. calcd. for $C_{15}H_9F_4N_3S$: C, 53.10; H, 2.67; N, 12.38. Found: C, 53.08; H, 2.63; N, 12.55.

4-(4-trifluoromethylphenyl)-5-(4-fluorophenyl)-2,4-dihydro-3*H*-1,2,4-triazole-3-thione (**16ct**). Yield: 80%. M.p. 282-284°C. ^{1}H-NMR (DMSO-d6) δ (ppm): 7.23-7.39 (m, 4H, ArH); 7.51 (d, 2H, ArH, *J* = 8.4 Hz); 8.03 (d, 2H, ArH, *J* = 8.4 Hz); 14.23 (s, 1H, NH). Anal. calcd. for $C_{15}H_9F_4N_3S$: C, 53.10; H, 2.67; N, 12.38. Found: C, 53.11; H, 2.54; N, 12.43.

List of 466 descriptors used in QSAR modeling:

molecular weight [mass_au]
log P
length
width
length/width
depth
width/depth
log P/width/depth
box volume [angstrom^3]
log P/box volume
box area [angstrom^2]
log P/box area
box cross section
log P/box cross section
H-bond donor count
H-bond acceptor count
total accessible surface area [angstrom^2]
nonpolar area
rotatable bond count
rotatable bond count nonterminal
all count
Hydrogen count
Carbon count
Oxygen count
Fluorine count
Chlorine count
Bromine count
all bond count
single bond count
Csp^3 bonded to 2 C
Csp^3 bonded to 1 C
Csp^2 bonded to 3 C
Csp^2 bonded to 2 C
amide count

sec-amine count
tertiary-amine count
methyl count
methylene count
ring count all
ring count all aromatic
ring count all nonaromatic
ring count 5 member
ring count nonaromatic 5
ring count 6 member
ring count aromatic 6
ring size smallest
energy dielectric [kcal/mol]
heat of formation [kcal/mol]
highest partial charge on H
highest partial charge on donatable H
lowest partial charge on free H acceptor
lowest partial charge on O
lowest partial charge on N
highest partial charge on C
lowest partial charge on C
second highest partial charge on H
third highest partial charge on H
highest partial charge on N
dipole moment from partial charges [debye]
highest electrophilic susceptibility
highest nucleophilic susceptibility
highest radical susceptibility
highest electrophilic susceptibility on C
highest nucleophilic susceptibility on C
highest radical susceptibility on C
highest electrophilic susceptibility on N
highest nucleophilic susceptibility on N
highest radical susceptibility on N
highest electrophilic susceptibility on O
highest nucleophilic susceptibility on O
highest radical susceptibility on O
highest electrophilic susceptibility on H
highest nucleophilic susceptibility on H
highest radical susceptibility on H
HOMO energy [eV]

LUMO energy [eV]
HOMO-LUMO gap
dipole moment [debye]
solvent accessible surf area [angstrom^2]
polarizability [angstrom^3]
highest partial charge
lowest partial charge
total positive partial charge
total negative partial charge
highest partial charge/total positive partial charge
lowest partial charge/total negative partial charge
partial positive surface area
partial positive surface area*total positive partial charge
partial negative surface area
partial negative surface area*total negative partial charge
partial positive surface area-partial negative surface area
partial positive surface area-partial negative surface area-partial negative surface area*total negative partial charge
partial positive surface area/total accessible surface area
partial negative surface area/total accessible surface area
partial positive surface area*total positive partial charge/total accessible surface area
partial negative surface area*total negative partial charge/total accessible surface area
abs(charge) weighted area
charge weighted area
charge weighted polar area
charge weighted nonpolar area
atomic charge weighted positive area
atomic charge weighted negative area
atomic charge weighted positive area-atomic charge weighted negative area
atomic charge weighted positive area/total accessible surface area
atomic charge weighted negative area/total accessible surface area
hydrogen donor partial surface area
hydrogen donor partial surface area/total accessible surface area
high charge partial surface area
high charge partial surface area/total accessible surface area
high positive charge partial surface area
high positive charge partial surface area/total accessible surface area
high negative charge partial surface area
high negative charge partial surface area/total accessible surface area
electrophilic weighted area
nucleophilic weighted area

radical weighted area
high electrophilic partial area
high nucleophilic partial area
high radical partial area
hydrophobic dipole
hydrophobicity weighted area
hydrophobicity weighted positive area
hydrophobicity weighted negative area
hydrophobicity weighted positive area/total accessible surface area
hydrophobicity weighted negative area/total accessible surface area
nonpolar area/accessible area
width>7
width>8
width>9
width>10
width>11
length>7
length>8
length>9
length>10
length>11
length>12
length>13
length>14
length>15
length>16
length>17
length>18
molecular weight>240
molecular weight>260
molecular weight>280
molecular weight>300
molecular weight>320
molecular weight>340
molecular weight>360
molecular weight>380
molecular weight>400
log P/MW
length/MW
width/MW
length/width/MW

depth/MW

width/depth/MW

log P/width/depth/MW

box volume/MW

box area/MW

box cross section/MW

H-bond donor count/MW

H-bond acceptor count/MW

total accessible surface area/MW

nonpolar area/MW

molecule count/MW

rotatable bond count/MW

rotatable bond count nonterminal/MW

all count/MW

Hydrogen count/MW

Carbon count/MW

Nitrogen count/MW

Oxygen count/MW

Fluorine count/MW

Sulfur count/MW

Chlorine count/MW

Bromine count/MW

all bond count/MW

single bond count/MW

double bond count/MW

Csp^3 bonded to 2 C/MW

Csp^3 bonded to 1 C/MW

Csp^2 bonded to 3 C/MW

Csp^2 bonded to 2 C/MW

Csp^2 bonded to 1 C/MW

amide count/MW

sec-amine count/MW

tertiary-amine count/MW

methyl count/MW

methylene count/MW

ring count all/MW

ring count all aromatic/MW

ring count all nonaromatic/MW

ring count 5 member/MW

ring count nonaromatic 5/MW

ring count 6 member/MW

ring count aromatic 6/MW
ring size smallest/MW
ring size largest/MW
energy dielectric/MW
heat of formation/MW
highest partial charge on H/MW
highest partial charge on donatable H/MW
lowest partial charge on free H acceptor/MW
lowest partial charge on O/MW
lowest partial charge on N/MW
highest partial charge on C/MW
lowest partial charge on C/MW
second highest partial charge on H/MW
third highest partial charge on H/MW
highest partial charge on N/MW
dipole moment from partial charges/MW
highest electrophilic susceptibility/MW
highest nucleophilic susceptibility/MW
highest radical susceptibility/MW
highest nucleophilic susceptibility on C/MW
highest radical susceptibility on C/MW
highest nucleophilic susceptibility on N/MW
highest nucleophilic susceptibility on O/MW
HOMO energy/MW
LUMO energy/MW
HOMO-LUMO gap/MW
dipole moment/MW
solvent accessible surf area/MW
polarizability/MW
highest partial charge/MW
lowest partial charge/MW
total positive partial charge/MW
total negative partial charge/MW
molecular weight^2
log P^2
length^2
width^2
length/width^2
depth^2
width/depth^2
log P/width/depth^2

box volume^2

box area^2

box cross section^2

log P/box cross section^2

H-bond donor count^2

H-bond acceptor count^2

total accessible surface area^2

nonpolar area^2

rotatable bond count^2

rotatable bond count nonterminal^2

all count^2

Hydrogen count^2

Carbon count^2

Fluorine count^2

all bond count^2

single bond count^2

Csp^3 bonded to 2 C^2

Csp^3 bonded to 1 C^2

Csp^2 bonded to 3 C^2

Csp^2 bonded to 2 C^2

methylene count^2

ring count all^2

ring count all aromatic^2

ring count 6 member^2

ring count aromatic 6^2

ring size smallest^2

energy dielectric^2

heat of formation^2

highest partial charge on H^2

highest partial charge on donatable H^2

lowest partial charge on free H acceptor^2

lowest partial charge on O^2

lowest partial charge on N^2

highest partial charge on C^2

lowest partial charge on C^2

second highest partial charge on H^2

third highest partial charge on H^2

highest partial charge on N^2

dipole moment from partial charges^2

highest electrophilic susceptibility^2

highest nucleophilic susceptibility^2

highest radical susceptibility^2
highest electrophilic susceptibility on C^2
highest nucleophilic susceptibility on C^2
highest radical susceptibility on C^2
highest nucleophilic susceptibility on N^2
highest radical susceptibility on N^2
highest nucleophilic susceptibility on O^2
highest radical susceptibility on O^2
HOMO energy^2
LUMO energy^2
HOMO-LUMO gap^2
dipole moment^2
solvent accessible surf area^2
polarizability^2
ln(molecular weight)
ln(log P)
ln(length)
ln(width)
ln(length/width)
ln(depth)
ln(width/depth)
ln(log P/width/depth)
ln(box volume)
ln(log P/box volume)
ln(box area)
ln(log P/box area)
ln(box cross section)
ln(log P/box cross section)
ln(H-bond donor count)
ln(H-bond acceptor count)
ln(total accessible surface area)
ln(nonpolar area)
ln(rotatable bond count)
ln(rotatable bond count nonterminal)
ln(all count)
ln(Hydrogen count)
ln(Carbon count)
ln(Fluorine count)
ln(all bond count)
ln(single bond count)
ln(Csp^2 bonded to 3 C)

ln(Csp^2 bonded to 2 C)

ln(ring count all)

ln(ring count all aromatic)

ln(ring count 6 member)

ln(ring count aromatic 6)

ln(ring size smallest)

ln(highest partial charge on H)

ln(highest partial charge on donatable H)

ln(highest partial charge on C)

ln(second highest partial charge on H)

ln(third highest partial charge on H)

ln(dipole moment from partial charges)

ln(highest electrophilic susceptibility)

ln(highest nucleophilic susceptibility)

ln(highest radical susceptibility)

ln(highest electrophilic susceptibility on C)

ln(highest nucleophilic susceptibility on C)

ln(highest radical susceptibility on C)

ln(highest electrophilic susceptibility on N)

ln(highest nucleophilic susceptibility on N)

ln(highest radical susceptibility on N)

ln(highest electrophilic susceptibility on H)

ln(highest nucleophilic susceptibility on H)

ln(highest radical susceptibility on H)

ln(HOMO-LUMO gap)

ln(dipole moment)

ln(solvent accessible surf area)

ln(polarizability)

1.0/molecular weight

1.0/log P

1.0/length

1.0/width

1.0/length/width

1.0/depth

1.0/width/depth

1.0/log P/width/depth

1.0/box volume

1.0/log P/box volume

1.0/box area

1.0/log P/box area

1.0/box cross section

1.0/log P/box cross section
1.0/H-bond donor count
1.0/H-bond acceptor count
1.0/total accessible surface area
1.0/nonpolar area
1.0/rotatable bond count
1.0/rotatable bond count nonterminal
1.0/ all count
1.0/ Hydrogen count
1.0/ Carbon count
1.0/ Fluorine count
1.0/all bond count
1.0/single bond count
1.0/Csp^2 bonded to 3 C
1.0/Csp^2 bonded to 2 C
1.0/ring count all
1.0/ring count all aromatic
1.0/ring count 6 member
1.0/ring count aromatic 6
1.0/ring size smallest
1.0/energy dielectric
1.0/heat of formation
1.0/highest partial charge on H
1.0/highest partial charge on donatable H
1.0/lowest partial charge on free H acceptor
1.0/lowest partial charge on N
1.0/highest partial charge on C
1.0/lowest partial charge on C
1.0/second highest partial charge on H
1.0/third highest partial charge on H
1.0/highest partial charge on N
1.0/dipole moment from partial charges
1.0/highest electrophilic susceptibility
1.0/highest nucleophilic susceptibility
1.0/highest radical susceptibility
1.0/highest electrophilic susceptibility on C
1.0/highest nucleophilic susceptibility on C
1.0/highest radical susceptibility on C
1.0/highest electrophilic susceptibility on N
1.0/highest nucleophilic susceptibility on N
1.0/highest radical susceptibility on N

1.0/highest electrophilic susceptibility on H

1.0/highest nucleophilic susceptibility on H

1.0/highest radical susceptibility on H

1.0/HOMO energy

1.0/LUMO energy

1.0/HOMO-LUMO gap

1.0/dipole moment

1.0/solvent accessible surf area

1.0/polarizability

sqrt(molecular weight)

sqrt(log P)

sqrt(length)

sqrt(width)

sqrt(length/width)

sqrt(depth)

sqrt(width/depth)

sqrt(log P/width/depth)

sqrt(box volume)

sqrt(log P/box volume)

sqrt(box area)

sqrt(log P/box area)

sqrt(box cross section)

sqrt(log P/box cross section)

sqrt(H-bond donor count)

sqrt(H-bond acceptor count)

sqrt(total accessible surface area)

sqrt(nonpolar area)

sqrt(rotatable bond count)

sqrt(rotatable bond count nonterminal)

sqrt(all count)

sqrt(Hydrogen count)

sqrt(Carbon count)

sqrt(Fluorine count)

sqrt(all bond count)

sqrt(single bond count)

sqrt(Csp^2 bonded to 3 C)

sqrt(Csp^2 bonded to 2 C)

sqrt(ring count all)

sqrt(ring count all aromatic)

sqrt(ring count 6 member)

sqrt(ring count aromatic 6)

sqrt(ring size smallest)
sqrt(highest partial charge on H)
sqrt(highest partial charge on donatable H)
sqrt(highest partial charge on C)
sqrt(second highest partial charge on H)
sqrt(third highest partial charge on H)
sqrt(dipole moment from partial charges)
sqrt(highest electrophilic susceptibility)
sqrt(highest nucleophilic susceptibility)
sqrt(highest radical susceptibility)
sqrt(highest electrophilic susceptibility on C)
sqrt(highest nucleophilic susceptibility on C)
sqrt(highest radical susceptibility on C)
sqrt(highest electrophilic susceptibility on N)
sqrt(highest nucleophilic susceptibility on N)
sqrt(highest radical susceptibility on N)
sqrt(highest electrophilic susceptibility on H)
sqrt(highest nucleophilic susceptibility on H)
sqrt(highest radical susceptibility on H)
sqrt(HOMO-LUMO gap)
sqrt(dipole moment)
sqrt(solvent accessible surf area)
sqrt(polarizability)

Docking binding poses:

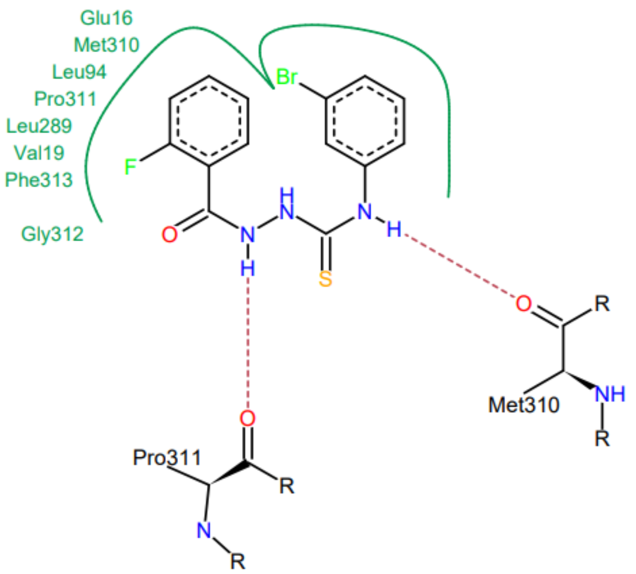

11a

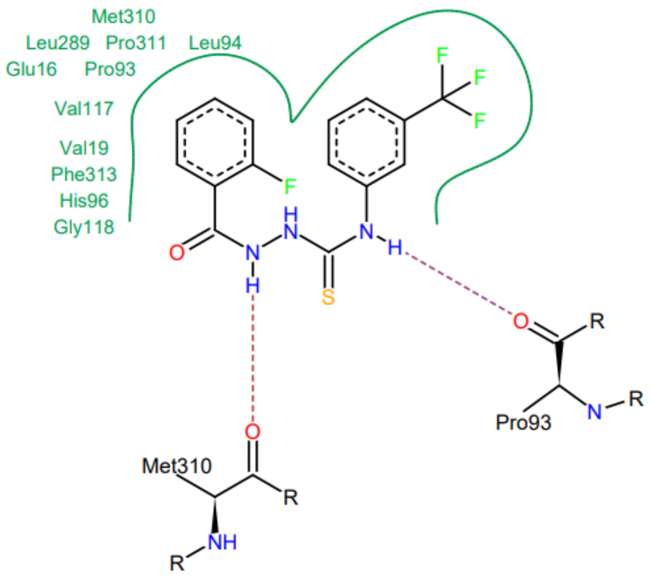

Met310
Leu289
Pro311
Leu94
Glu16
Pro93
Val117
Val19
Phe313
His96
Gly118
Met310
Pro93

15a

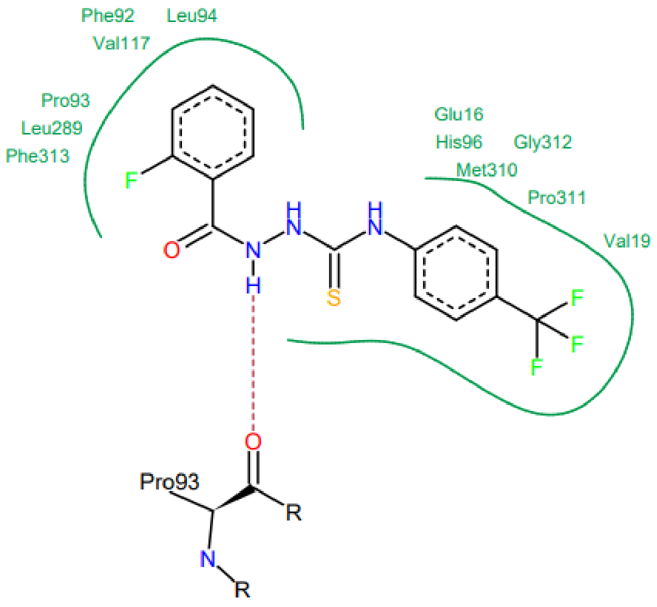

Phe92
Leu94
Val117
Pro93
Leu289
Phe313
Glu16
His96
Gly312
Met310
Pro311
Val19
Pro93

16a

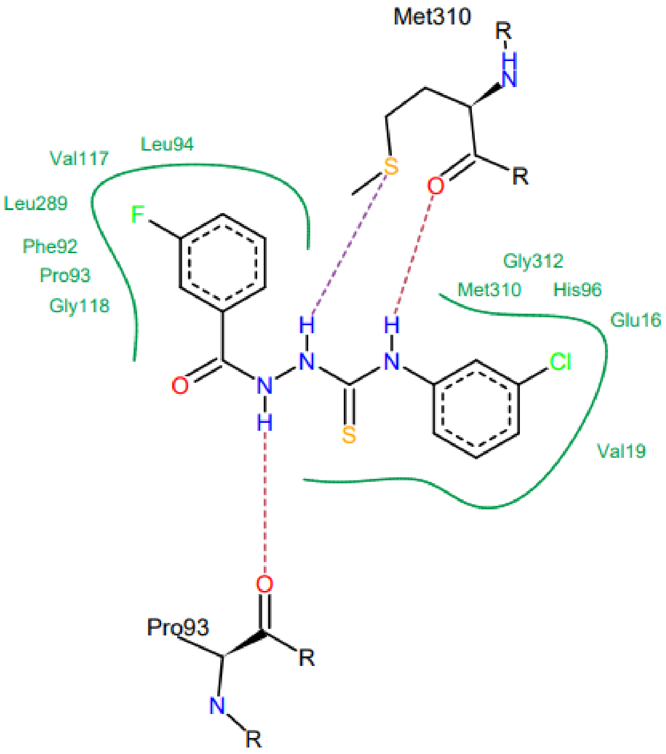

Met310
Val117
Leu94
Leu289
Phe92
Pro93
Gly118
Gly312
Met310
His96
Glu16
Val19
Pro93

9b

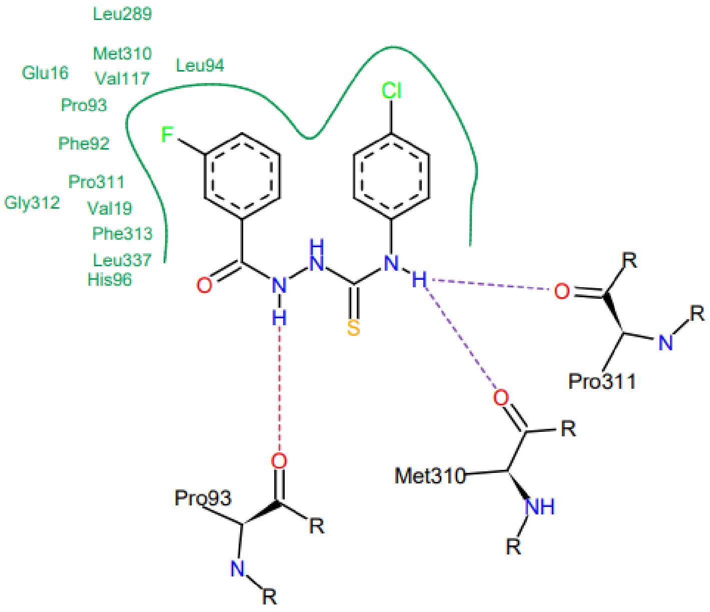

Leu289
Met310
Glu16
Val117
Leu94
Pro93
Phe92
Pro311
Gly312
Val19
Phe313
Leu337
His96
Pro311
Met310
Pro93

10b

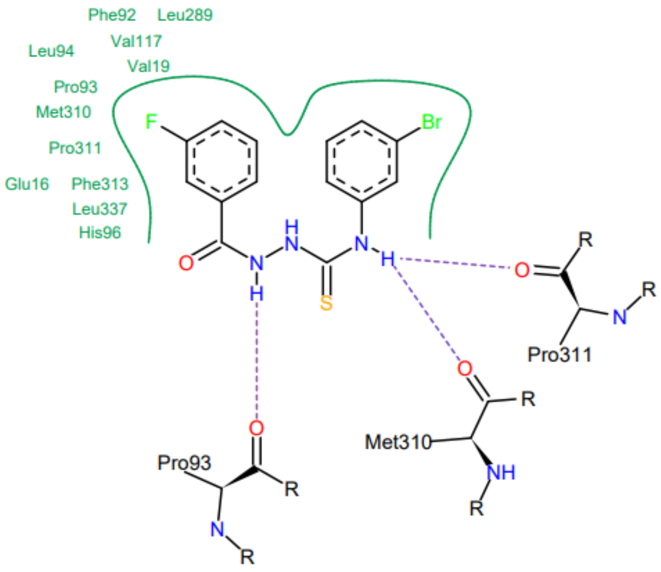
Phe92
Leu289
Leu94
Val117
Val19
Pro93
Met310
Pro311
Glu16
Phe313
Leu337
His96
F
Br
Pro311
Met310
Pro93

11b

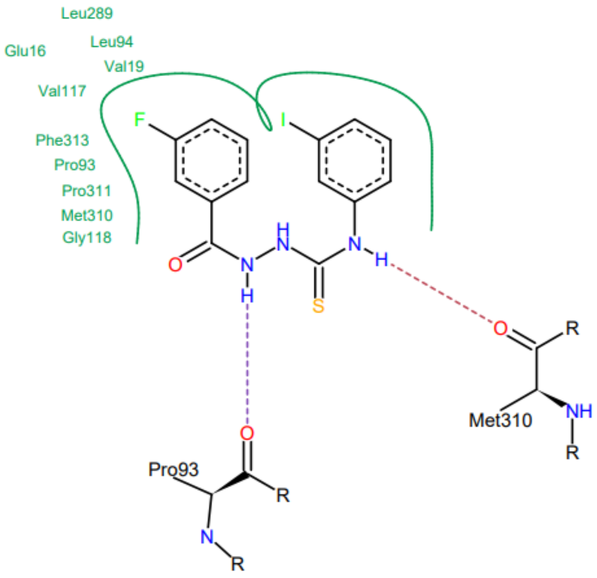
Leu289
Leu94
Glu16
Val19
Val117
Phe313
Pro93
Pro311
Met310
Gly118
F
I
Met310
Pro93

13b

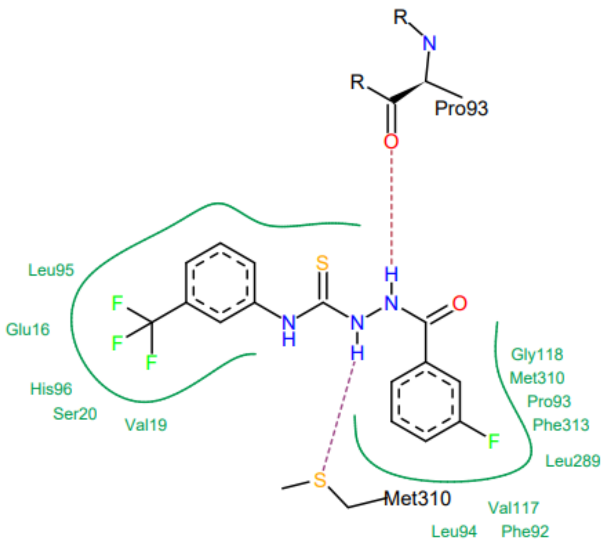
R
N
R
Pro93
O
Leu95
Glu16
His96
Ser20
Val19
F
F
F
S
N
H
N
H
H
N
O
Gly118
Met310
Pro93
Phe313
F
Leu289
S
Met310
Val117
Leu94
Phe92

15b

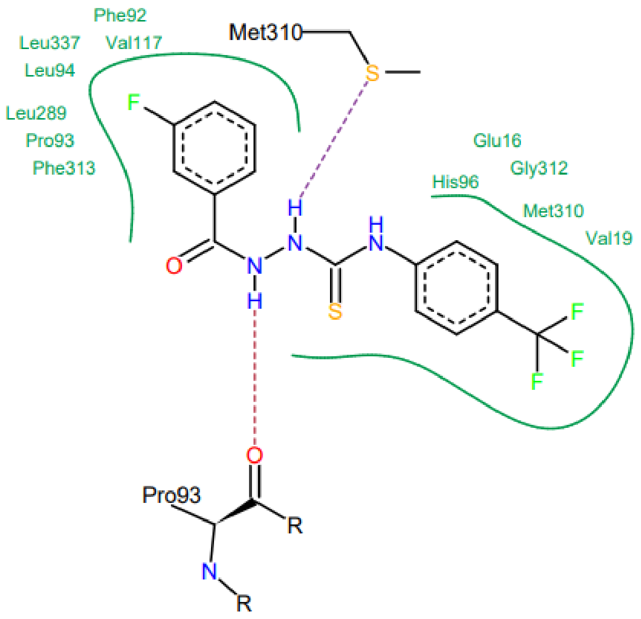
Phe92
Leu337
Val117
Leu94
Met310
S
Leu289
Pro93
Phe313
F
Glu16
Gly312
His96
Met310
Val19
H
N
H
N
O
N
H
S
F
F
F
O
Pro93
R
N
R

16b
